# Adiponectin is an endogenous anti-fibrotic mediator and therapeutic target

**DOI:** 10.1038/s41598-017-04162-1

**Published:** 2017-06-30

**Authors:** Roberta G. Marangoni, Yuri Masui, Feng Fang, Benjamin Korman, Gabriel Lord, Junghwa Lee, Katja Lakota, Jun Wei, Philipp E. Scherer, Laszlo Otvos, Toshimasa Yamauchi, Naoto Kubota, Takashi Kadowaki, Yoshihide Asano, Shinichi Sato, Warren G. Tourtellotte, John Varga

**Affiliations:** 1 Northwestern Scleroderma Program, Feinberg School of Medicine, Chicago, IL 60611 USA; 2 Department of Dermatology, University of Tokyo Graduate School of Medicine, Bunkyo-ku, Tokyo 113-8655 Japan; 3 Department of Preventive Medicine, Northwestern University Feinberg School of Medicine, Chicago, IL 60611 USA; 4 Department of Rheumatology, University Medical Centre Ljubljana, 1000 Ljubljana, Slovenia; 5 Touchstone Diabetes Center, Department of Internal Medicine, University of Texas Southwestern Medical Center, Dallas, TX 75390 USA; 6 Department of Biology, Temple University, Philadelphia, PA 19140 USA; 7 Department of Diabetes and Metabolic Diseases, University of Tokyo Graduate School of Medicine, Bunkyo-ku, Tokyo 113-8655 Japan; 8 Department of Pathology and Neurology, Northwestern University Feinberg School of Medicine, Chicago, IL 60611 USA

## Abstract

Skin fibrosis in systemic sclerosis (SSc) is accompanied by attrition of dermal white adipose tissue (dWAT) and reduced levels of circulating adiponectin. Since adiponectin has potent regulatory effects on fibroblasts, we sought to assess adiponectin signaling in SSc skin biopsies, and evaluate fibrosis in mice with adiponectin gain- and loss-of-function mutations. Furthermore, we investigated the effects and mechanism of action of agonist peptides targeting adiponectin receptors *in vitro* and *in vivo*. We found that adiponectin pathway activity was significantly reduced in a subset of SSc skin biopsies. Mice lacking adiponectin mounted an exaggerated dermal fibrotic response, while transgenic mice with constitutively elevated adiponectin showed selective dWAT expansion and protection from skin and peritoneal fibrosis. Adiponectin receptor agonists abrogated *ex vivo* fibrotic responses in explanted normal and SSc fibroblasts and in 3D human skin equivalents, in part by attenuating focal adhesion complex assembly, and prevented and reversed experimentally-induced organ fibrosis in mice. These results implicate aberrant adiponectin pathway activity in skin fibrosis, identifying a novel function for this pleiotropic adipokine in regulation of tissue remodeling. Restoring adiponectin signaling in SSc patients therefore might represent an innovative pharmacological strategy for intractable organ fibrosis.

## Introduction

Systemic sclerosis (SSc) is a chronic fibrotic disease with no approved disease-modifying therapies and high mortality rates that have remained unchanged for decades^1^. Fibrosis is characterized by myofibroblast persistence within lesional tissues, with sustained production of collagens and other extracellular matrix (ECM) components, and increased tissue stiffness leading to disruption of organ architecture^2^. While extracellular cues such as transforming growth factor-ß (TGF-ß) and Wnt ligands have been implicated as triggers for fibroblast activation in SSc, the mechanisms driving persistent fibrosis remain incompletely understood, hindering the development of effective therapies^3^.

Dermal fibrosis, the hallmark of SSc, is commonly accompanied by attrition and diasappearance of the dermal adipose layer. This layer, recently designated dermal white adipose tissue (dWAT), represents a distinct adipose depot that lies within the skin and has unique features, developmental origins, and functional roles distinguishing it from other WAT depots^4^. Multiple lines of evidence underscore the critical contributions of dWAT to dermal physiology, including hair cycle regulation, wound healing, thermal insulation and anti-microbial protection^5^. These vital skin functions are impaired in mice with genetically or pharmacologically disrupted dWAT homeostasis^5–8^. Together, these observations suggest that attrition of dWAT in SSc, as well as in distinct disease models, might play a pathogenic role in skin fibrosis.

Attrition of dWAT in SSc is associated with loss of adipocytes^9^. Adipogenesis is controlled by PPAR-gamma (PPAR-γ), a pleiotropic nuclear receptor involved in obesity, diabetes and cancer, and increasingly implicated in inflammation and fibrosis^10^. Pharmacological ligands of PPAR-γ attenuate fibroblast activation and myofibroblast differentiation *in vitro* ^11^, and ameliorate organ fibrosis while preventing dWAT attrition *in vivo* ^12–15^. Conversely, PPAR-γ ablation is associated with impaired adipogenesis and exaggerated fibrotic responses^10, 16, 17^. The anti-fibrotic effects of PPAR-γ are partially mediated by adiponectin, a 30 kDa secreted adipokine abundant in the circulation^18^. While adiponectin is secreted almost exclusively by adipocytes, its concentrations in the circulation are inversely proportional to the adipose mass, a paradox reflecting the fact that it is adipocyte quality, rather than quantity, that determines net adiponectin production^18–20^. The pleiotropic activities of adiponectin are mediated through the transmembrane receptors AdipoR1 and AdipoR2 and intracellular pathways involving adenosine monophosphate (AMP)-activated protein kinase (22). Circulating adiponectin levels are reduced in patients with diffuse cutaneous SSc and show negative correlation with disease activity, severity and duration^21–24^. Adiponectin attenuates fibroblast activation, and reverses the activated phenotype of SSc fibroblasts^25, 26^. We therefore sought to characterize adiponectin pathway activity in SSc, and determine the role and mechanism of action of adiponectin in mouse models of fibrosis. Using genetic and pharmacologic approaches, we show that adiponectin acts as an endogenous anti-fibrotic mediator that is down-regulated in SSc. Loss of adiponectin signaling exacerbates, while augmenting it mitigates, skin fibrosis in mice. Moreover, synthetic peptides targeting AdipoR have potent anti-fibrotic effects *in vitro* and *in vivo*. Taken together, these findings implicate adiponectin deficiency as a pathogenic driver of persistent skin fibrosis, and provide evidence that restoring adiponectin signaling may represent a promising novel therapeutic strategy for mitigating fibrosis in SSc.

## Results

### Deregulated adiponectin signaling in SSc skin biopsies

**Figure 1:**
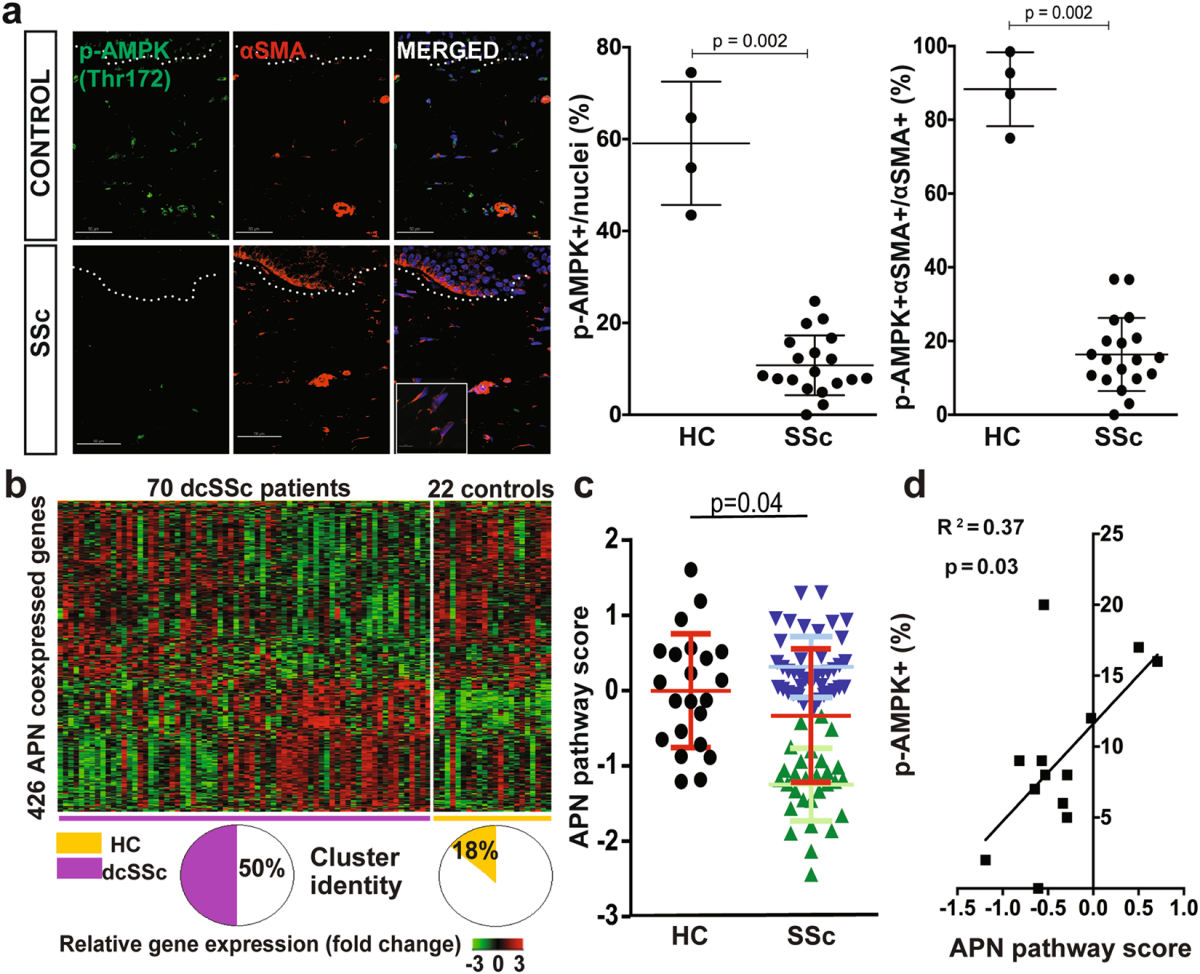
Attenuated adiponectin signaling in SSc skin biopsies. (**a**) Left, immunofluorescence using antibodies to phospho-AMP-activated protein kinase (Thr172) (p-AMPK; green) and α-smooth muscle actin (αSMA, red); nuclei stained with DAPI (blue). Skin biopsies from SSc patients (n = 20) and age-matched healthy controls (n = 4) were examined. Dotted lines indicate the border between the epidermis and dermis. Representative photomicrographs; scale bar = 50 µm. Inset illustrating interstitial myofibroblasts within lesional dermis that are p-AMPK negative, scale bar = 10 µm. Right, quantification of p-AMPK-positive cells within the dermis. Results are shown as pAMPK^+^ cells/total nuclei, and p-AMPK/α-SMA double-positive cells/αSMA^+^ cells. Values are means ± SD from three high-power fields in each biopsy. (**b**) Unsupervised cluster analysis. Heatmap demonstrating altered expression of genes (432) significantly correlated with adiponectin mRNA levels (r > 0.4, p < 0.005) measured in 70 SSc (left) and 20 control (right) skin biopsies. Red (green) color indicates higher (lower) levels of gene expression. Note that 37/70 subjects map to a subset with distinctly differentially regulated expression pattern. **(c)** Adiponectin signaling scores (described in Methods) were measured in skin biopsy transcriptome dataset (GSE49332). In addition to the significant difference between SSc and controls (red bars, mean ± SD, p = 0.04), note bimodal distribution in SSc, with 41 patients (blue) demonstrating normal adiponectin pathway scores and 29 (green) showing markedly decreased pathway scores. **(d)** Adiponectin pathway activation scores are significantly correlated with p-AMPK expression.

We showed previously that levels of circulating adiponectin are reduced in patients with SSc^23^. Since dWAT attrition might account for reduced adiponectin, we speculated that adiponectin signaling within the lesional skin might also be altered in SSc. To directly assess adiponectin activity, we measured tissue levels of phosphorylated AMP-activated protein kinase (pAMPK), a downstream mediator of AdipoR1-dependent responses that is a marker for adiponectin activity^27^. By immunofluorescence, we found that dermal levels of cellular pAMPK were significantly reduced in SSc skin biopsies (n = 19) compared to healthy controls (n = 4; p < 0.01) (Fig. 1; Supplementary Table 1). Double-label immunofluorescence showed that a majority of α-SMA-positive interstitial myofibroblasts in the lesional dermis had low or absent pAMPK, suggesting a causal role for diminished cellular adiponectin signaling in myofibroblast accumulation and persistence.

We next sought to evaluate the expression of adiponectin-regulated and co-regulated genes in skin by querying our previously reported, publicly available microarray datasets from 70 SSc patients and 22 healthy controls (GSE76886)^28^. First, we used genes significantly correlated with adiponectin expression to define a 432-gene adiponectin co-regulated (synexpression) gene set (r > 0.4, p < 0.005; Supplementary Table 2)^29^. Unsupervised hierarchical clustering using this synexpression gene set segregated SSc skin biopsies into a normal-like subset and a subset showing reduced levels of the synexpression set (Fig. 1b). Fifty percent of SSc biopsies (35/70) mapped to the reduced adiponectin synexpression subset, while 82% of healthy control biopsies (18/22) mapped to the normal-like subset. There was significant enrichment of SSc vs control disease status in the reduced adiponectin subset (OR 4.5, 1.5–13.8, p = 0.008). Gene ontology (GO) analysis revealed significant over-representation of processes related to immunity, epidermal function, cellular processes, cell metabolism and collagen organization in the adiponectin synexpression gene set **(**Supplementary Table 3 **)**. A complementary analysis was used to define an “adiponectin-regulated gene signature” by selecting the top 20 up- and top 20 down-regulated genes from a transcriptome dataset from adiponectin-treated cells (GSE49332)^30^. A pathway score was generated using the 29 genes from this list that were present in the SSc biopsy dataset (Supplementary Table 4)^31^. Adiponectin pathway scores were reduced in the SSc skin biopsies (p = 0.04), and showed significant correlation with cellular pAMPK levels (p = 0.03) within the same biopsies (Fig. 1c,d). Consistent with the analysis using synexpression subsets, a subpopulation of SSc biopsies (adiponectin^low^, n = 29) showed decreased adiponectin pathway activity (defined as pathway score ≤ 95% C.I. of the mean score of the controls), whereas 41 SSc biopsies (adiponectin^normal^) showed adiponectin signatures comparable to controls. There were no significant differences between these two SSc subsets in terms of age, sex, body mass index, disease duration or MRSS; however, the limited amount of clinical information in the publicly available datasets precluded in-depth comparisons. The divergence of adiponectin signaling activity between the two skin biopsy subsets reflects the molecular heterogeneity of the SSc^32^. Taken together, these analyses provide evidence for significantly impaired adiponectin signaling in SSc, while also highlighting the molecular heterogeneity of SSc skin biopsies. These intriguing findings prompted us to assess adiponectin as a potential driver of fibrosis.

### Exaggerated skin fibrosis in adiponectin-null mice

**Figure 2:**
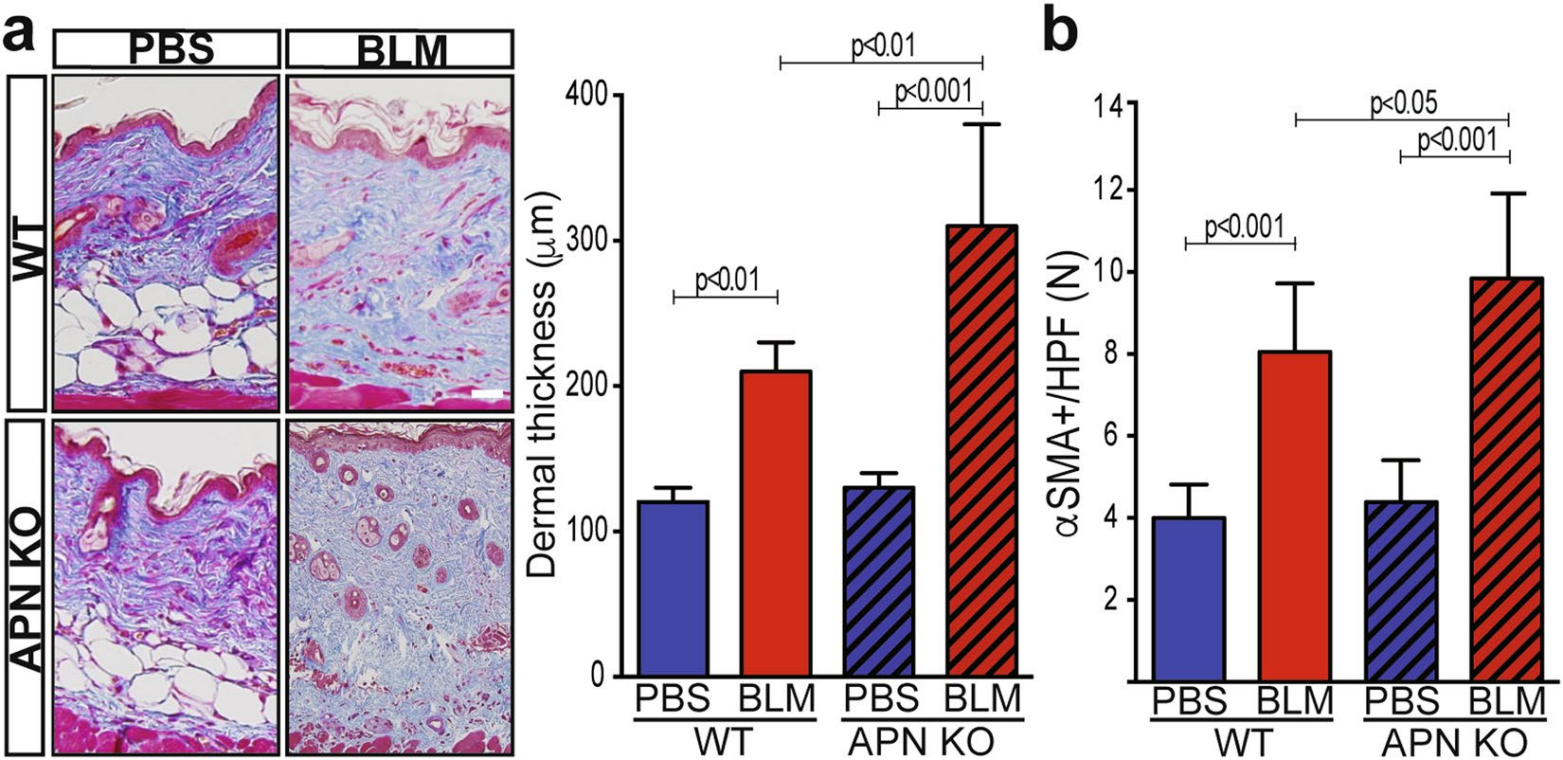
Adiponectin knockout mice develop exaggerated dermal fibrosis upon bleomycin treatment. Adiponectinknockout (APN KO) and wildtype (WT) mice received daily s.c. injections of bleomycin (BLM) or PBS (control) for 14 days and lesional skin was harvested for analysis. (**a**) Left panel, Masson’s Trichrome stain; representative images; scale bar = 100 µm. Right panel, dermal thickness, mean ± SD from seven determinations from 4–8 mice per experimental group. (**b**) αSMA^+^ cells were counted in five hpf/slide. Results are means ± SD.

To assess the contribution of adiponectin in skin fibrosis, both gain- and loss-of-function experiments were performed. Chronic treatment of adiponectin KO mice with bleomycin (BLM) resulted in exacerbation of skin fibrosis in compared to identically-treated wildtype mice, with significantly greater increase in collagen deposition (Fig. 2a **)** and myofibroblast accumulation within the fibrotic dermis **(**Fig. 2b **)**. Endogenous adiponectin thus appears to have an important role in dermal homeostasis by limiting fibroblast activation.

### Attenuated skin fibrosis and dWAT attrition in adiponectin-overexpressing transgenic mice

**Figure 3:**
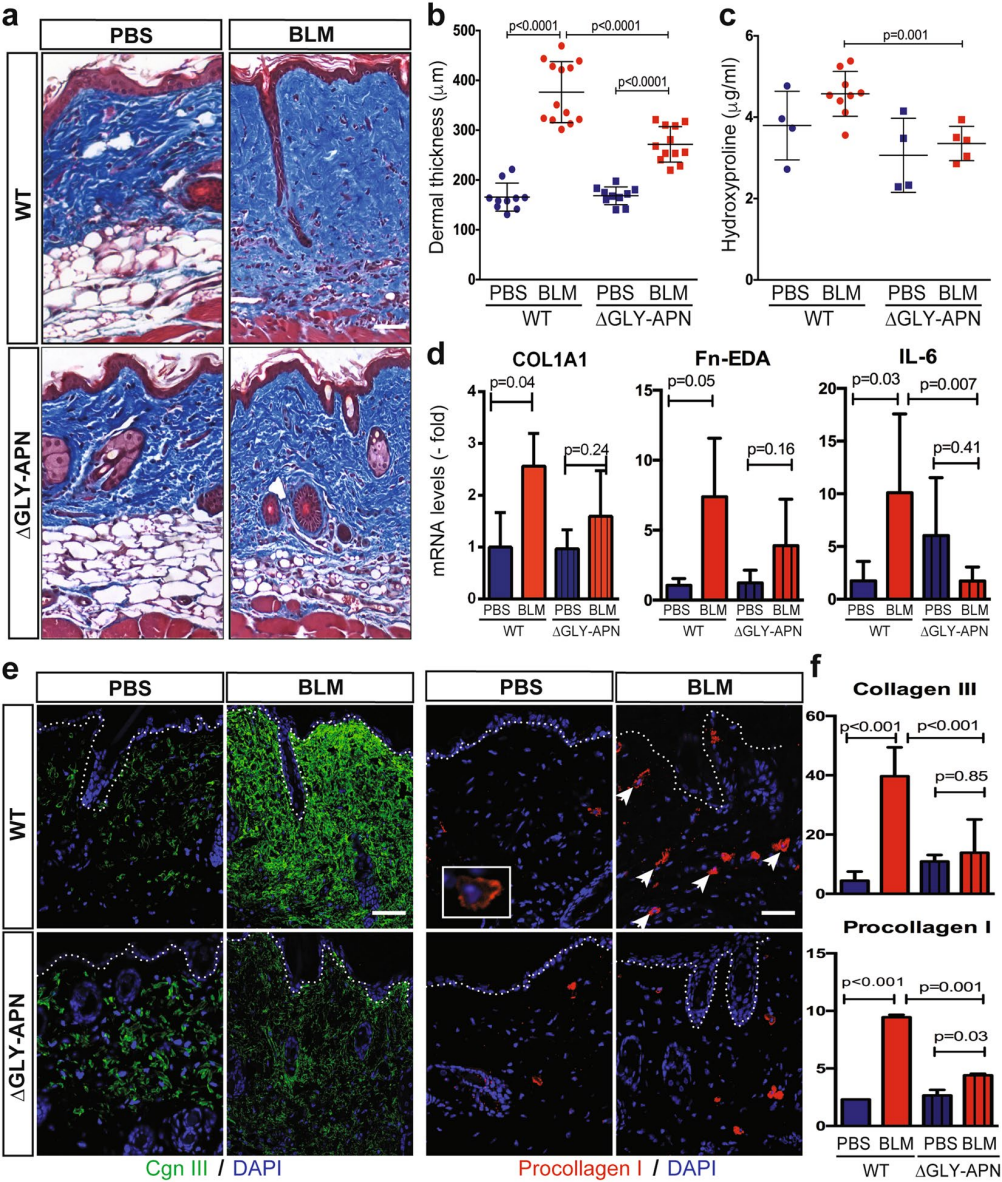
ΔGLY-APN transgenic mice have reduced dermal fibrosis and attrition of dermal white adipose tissue in response to bleomycin treatment. Eight to twelve week-old ΔGLY-APN and wildtype (WT) mice received s.c. injections of bleomycin (BLM) or PBS (control) daily for 14 days, and skin was harvested at day 2. **(a)** Masson’s Trichrome stain (representative images); scale bar = 100 µm. **(b)** Dermal thickness. Mean ± SD from three independent experiments (n = 10–13/group). (**c)** Hydroxyproline assays. Results are the means ± SD of triplicate determinations (4 mice per group for control, 5–9 mice/group for BLM). **(d)** RNA was analyzed by qPCR. Results, normalized to GAPDH, are mean ± SD of triplicate determinations (n = 3/group for control, n = 5/group for BLM). **(e)** Immunofluorescence using antibodies to type III collagen (left panels, green) or procollagen I (right panels, red). Representative photomicrographs. Dotted lines indicate epidermal/dermal junction; arrows indicate procollagen I-positive cells within the dermis. Scale bars = 100 µm. **(f)** Immunopositive cells were counted. Results are means ± SD from three hpf/slide (n = 3 mice/group). Three independently repeated experiments yielded consistent results.

We next investigated whether boosting endogenous adiponectin could modulate skin fibrosis. ΔGLY-adiponectin (ΔGLY-APN) transgenic mice produce a truncated adiponectin with deletion of the 13–22 Gly-X-Y repeats in the collagenous domain^33^. Mutant adiponectin promotes release of endogenous adiponectin by preventing its intracellular degradation^33^. At 8–12 weeks of age, ΔGLY-APN mice showed 2–3-fold higher levels of circulating adiponectin than age-matched wildtype mice (p < 0.01, Supplementary Fig. 1a), and selective expansion of dWAT (8–11 cell layers compared to 4–5 cell layers in wildtype mice) (Fig. 3a). Importantly, BLM-induced increase in dermal thickness, collagen deposition and dWAT attrition were all significantly attenuated in ΔGLY-APN transgenic mice (Fig. 3a–c). Levels of circulating adiponectin showed negative correlation with both dermal thickness (r = −0.747, p < 0.0001) and collagen content (r = −0.586, p = 0.027) (Supplementary Fig. 1b). In addition, BLM-induced increase in IL-6 and fibrotic gene expression, the proportions of collagen-producing activated fibroblasts, and collagen accumulation within the lesional skin were all significantly attenuated in ΔGLY-APN mice (Fig. 3d–f and Supplementary Fig. 2). By controlling assembly of focal adhesion complexes, focal adhesion kinase (FAK) plays a crucial role in myofibroblast differentiation, and is implicated in SSc skin fibrosis^34^. Immunofluorescence analysis showed that FAK phosphorylation within lesional skin myofibroblasts was attenuated in ΔGLY-APN transgenic mice (Supplementary Fig. 3).

Since TGF-β is widely recognized to play a fundamental pathogenic role in SSc fibrosis, we utilized a model of skin fibrosis induced by local expression of a constitutively active TGFβ (TGFß1^223/225^). Increased dermal thickness, collagen deposition and expression of fibrosis-related genes induced by Ad-TGFβ1 were all attenuated in ΔGLY-APN transgenic mice **(**Supplementary Fig. 4a–c **)**. To examine the effect of adiponectin on extra-cutaneous fibrosis, we employed a model of peritoneal fibrosis^35^. By 21 days of alternate-day i.p. injections of chlorhexidine gluconate (CG), wildtype mice developed marked increase in peritoneal membrane thickness (Supplementary Fig. 5). Strikingly, similarly-treated ΔGLY-APN mice showed substantial reduction of peritoneal fibrosis, cell proliferation and myofibroblast accumulation. Together these findings identify a protective effect of endogenous adiponectin in distinct models of organ fibrosis.

### Agonist peptides targeting AdipoR inhibit fibrotic response *in vitro*

**Figure 4:**
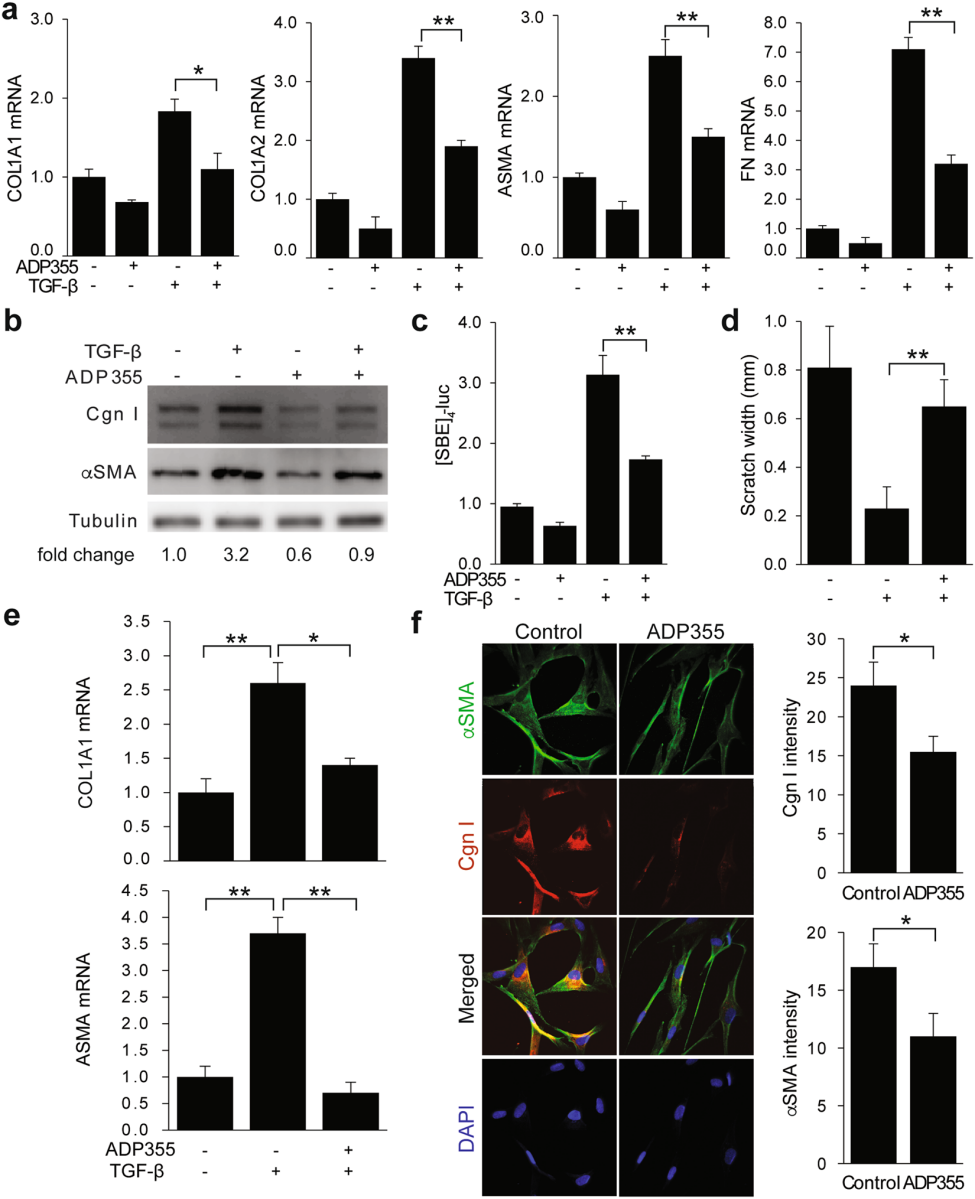
ADP355 inhibits fibrotic responses in normal and SSc skin fibroblasts. (**a–d**). Confluent cultures of normal dermal fibroblasts were preincubated with ADP355, followed by TGF-β2 for 24 h. (**a**) Total RNA was subjected to real-time qPCR. Results are mean ± SD of triplicate determinations. *p < 0.05, **p < 0.01. (**b**) Western blot of whole cell lysates. Representative results. Fold change in Type I collagen (Cgn I) levels relative to control is shown at bottom. (**c**) Fibroblasts were transiently transfected with [SBE]_4_-luc reporter plasmids, and cell lysates were assayed for their luciferase activities. Results are mean ± SD of triplicate determinations. **p < 0.001 (**d**) Scratch assays were performed and the widths determined after 48 h. Results are mean ± SEM of triplicate determinations at three randomly selected sites. (**e**) 3D human skin equivalents populated with normal fibroblasts were incubated in media with ADP355 and TGF-β2 for 6 days. RNA was isolated for qPCR. Results are mean ± SD of triplicate determinations *p < 0.05, **p < 0.001. (**f**) SSc skin fibroblasts (n = 5) incubated with ADP355 for 24 h were immunostained with antibodies to αSMA (green) or type I collagen (Cgn1, red), or stained with DAPI (blue). Left panels, representative immunofluorescence photomicrographs (original magnification × 400). Right panels, immunofluorescence intensity. Results are means ± SD from five randomly selected hpf.

**Figure 5:**
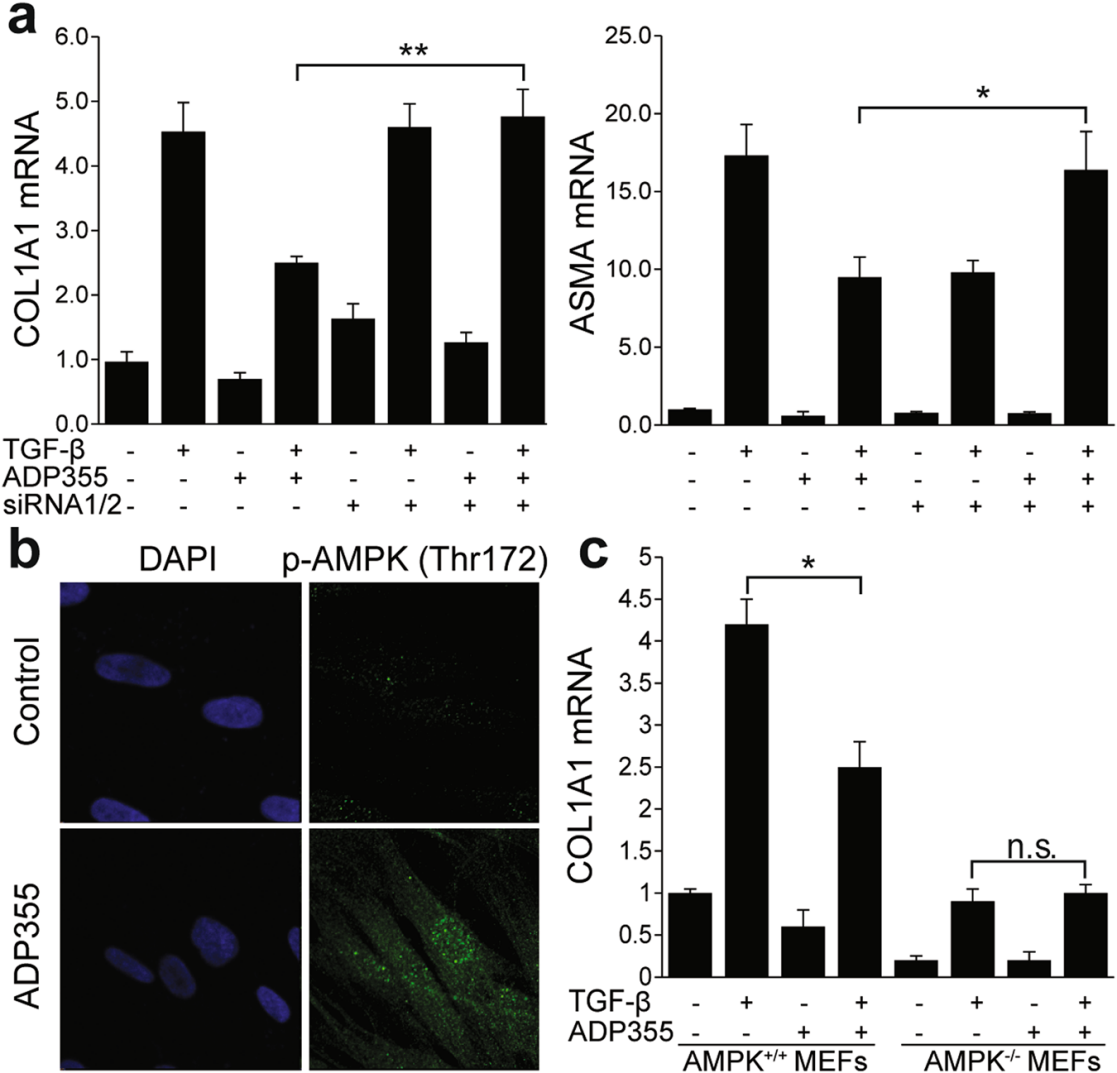
The anti-fibrotic effects of ADP355 are mediated by adiponectin receptor signaling and AMP protein kinase activation. Confluent cultures of human (**a,b**) or mouse (**c**) fibroblasts were incubated in media with ADP355 for 24 h (**a,c**) or 60 min (**b**). (**a**) Fibroblasts were transfected with AdipoR1/R2 siRNA (siR1/2), or scrambled control siRNA for 24 h prior to ADP355 and TGF-β. Results of qPCR shown as means ± SD of triplicate determinations. *p < 0.05, **p < 0.001. **(b)** Fibroblasts were immunostained with antibodies to phospho-AMP kinase (Thr172) (p-AMPK, green), or stained with DAPI (blue), and examined by confocal microscopy. Representative immunofluorescence photomicrographs. Original magnification x400. **(c)** Total RNA was examined by real-time qPCR. Results are the mean ± SD of triplicate determinations. *p < 0.05.

The complex quaternary structure of adiponectin, combined with its extreme insolubility and relatively short half-life, pose major impediments to physiologic replacement as a viable therapeutic strategy^36^. An alternate potential strategy is to mimic adiponectin biological activity using small molecules. High-throughput screening of a panel of 66 overlapping 10-amino acid peptides covering the entire globular domain of adiponectin (residues 105–254) identified ADP355 (AA149-166 at active site) as a potent AdipoR agonist^37^. This peptide was shown to have anti-tumor effects in AdipoR-positive cancer cell lines, and to reverse hypoadiponectinemia and loss of subcutaneous adipose tissue induced by HIV protease inhibitors^38^. Incubation of neonatal skin fibroblasts with ADP355 resulted in potent abrogation of COL1A1, COL1A2, ASMA, and fibronectin (FN) gene expression induced by TGF-ß (Fig. 4a,b). No effects on cell viability were observed with the concentrations of ADP355 used for these experiments. Comparable inhibitory effects of ADP355 were observed in adult skin fibroblasts (data not shown). Specific binding of ADP355 to fibroblasts was confirmed by *in vitro* competition binding experiments using labeled peptides (Supplementary Fig. 6a). In transient transfection assays, ADP355 abrogated the stimulation of [SBE]_4_-luc activity by TGF-ß, indicating that the negative regulatory effect involved disruption of canonical Smad pathways (Fig. 4c). Rapid Smad2/3 phosphorylation elicited by TGF-ß was partially reduced by ADP355 treatment of the fibroblasts (Supplementary Fig. 7). ADP355 abrogated the stimulation of fibroblast migration, and attenuated fibrotic responses in 3D human skin equivalents populated with normal skin fibroblasts, and mitigated constitutive fibrotic gene expression in unstimulated SSc fibroblasts (Fig. 4d–f). Adiponectin binding to AdipoR1 and AdipoR2 is required for its anti-fibrotic effects. Silencing of AdipoR1/R2 in normal fibroblasts (resulting in 80% decrease of AdipoR1 and R2 levels, data not shown), substantially reversed the inhibition of fibrotic gene expression by ADP355 (Fig. 5a). We previously showed that the anti-fibrotic activities of adiponectin were mimicked by the AMP-activated protein kinase (AMPK) activator AICAR, and were blocked by Compound C, implicating AMPK in this response^25^. In normal fibroblasts, ADP355 induced AMPK phosphorylation **(**Fig. 5b **)**, and failed to exert anti-fibrotic activity in AMPK-null MEFs, confirming an essential role of AMP-activated kinase in mediating these effects **(**Fig. 5c **)**. Focal adhesion complex assembly regulated by FAK has a critical role in myofibroblast activation by TGF-ß, and is implicated in fibrosis^34, 39, 40^. Adiponectin has been shown to promote focal adhesion complex disassembly in activated hepatic stellate cells^41^. While in normal skin fibroblasts TGF-ß augmented FAK phosphorylation (p-FAK Y397) and enhanced formation of focal adhesion complexes, pretreament with ADP355 substantially attenuated both FAK activation and focal adhesion complex assembly (Supplementary Fig. 8a–c).

### ADP355 attenuates skin fibrosis *in vivo*

**Figure 6:**
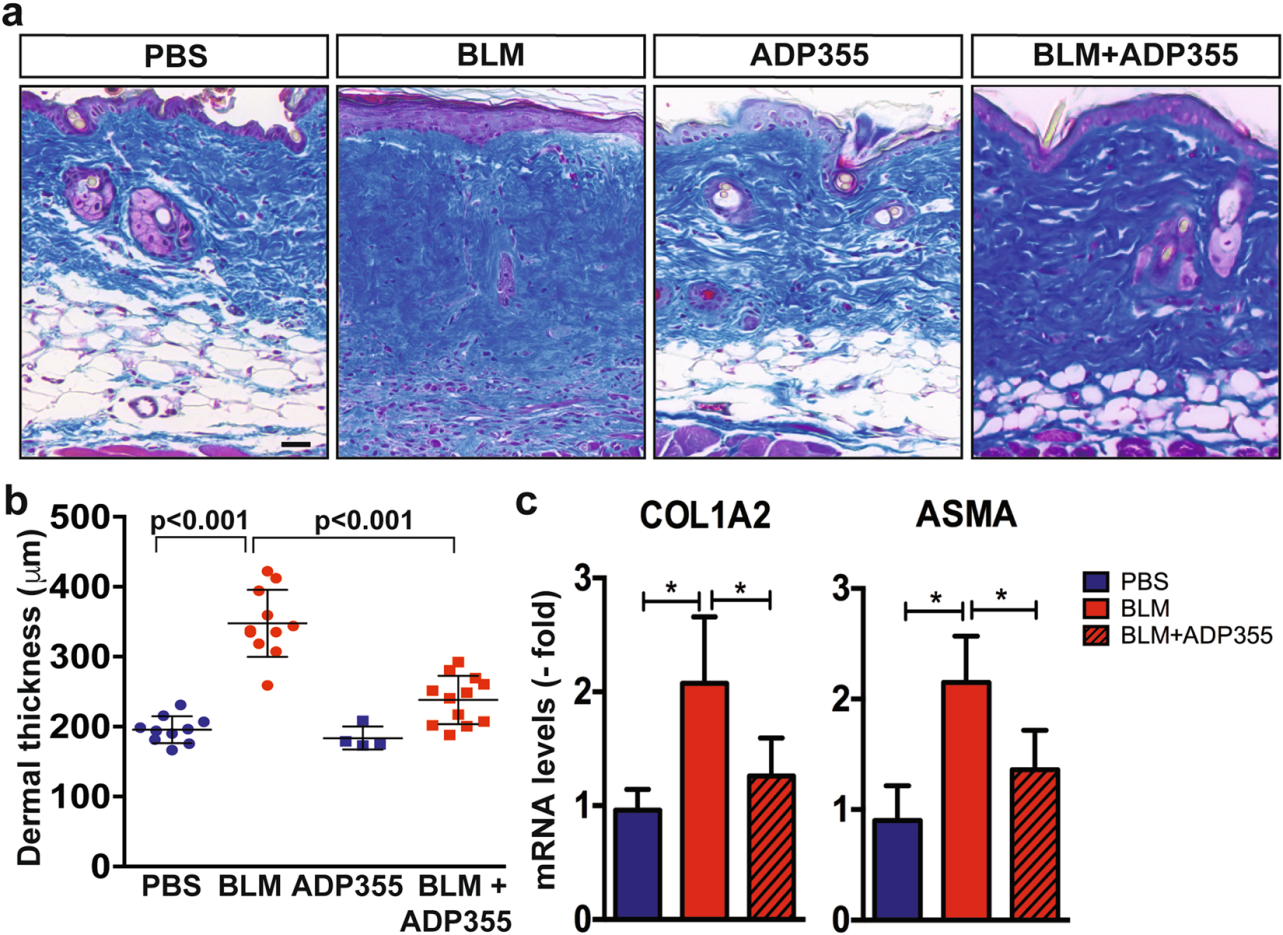
ADP355 treatment attenuates bleomycin-induced skin fibrosis in mice. C57BL6/J mice received bleomycin (BLM) or PBS via daily s.c. injections for 14 d, and daily i.p. injections of ADP355 or vehicle initiated concomitantly with BLM or PBS, and continued for 21 d, when lesional skin was harvested. (**a**) Masson’s Trichrome stain. Representative images; scale bar = 100 µm. (**b**) Dermal thickness. Results are means ± SD of triplicate determinations from two independent experiments (10–12 per group for BLM and BLM + ADP355, 4 mice/group for PBS or ADP355 alone). (**c**) RNA was analyzed by real-time qPCR. Results are the mean ± SD of 5 mice/group. *p < 0.05.

We next evaluated the effects of ADP355 treatment in mice. The peptide demonstrated excellent *ex vivo* stability, with detectable levels of intact peptide even after 30 min incubation with serum (data not shown). Intraperitoneal injection of labeled ADP355 i.p. led to its rapid (10 min) accumulation within the skin (Supplementary Fig. 6b). Chronic treatment with ADP355 (1 mg/kg/d i.p.) for up to 28 days was well tolerated, with no signs of toxicity. When initiated concurrently with BLM, ADP355 treatment significantly mitigated the increase in dermal thickness, collagen accumulation, fibrotic gene expression and dWAT attrition (Fig. 6), while by itself, ADP355 had no significant effect on dermal thickness or on circulating adiponectin levels **(**Fig. 6; and data not shown). Significant attenuation of skin fibrosis was consistently observed in multiple independent experiments with both low (0.2 mg/kg/d) and high (1 mg/kg/d) doses of ADP355. Importantly, ADP355 treatment initiated at day 10 of BLM reversed established dermal fibrosis (data not shown). Comparable anti-fibrotic effects were elicited in mice treated with ADP27, a 10-amino acid peptide analog of ADP355 consisting of the minimum active site of adiponectin (Supplementary Fig. 9a,b). Peptide treatment was associated with evidence of increased AMPK phosphorylation in skeletal muscle (Supplementary Fig. 9c).

## Discussion

The observation that dermal fibrosis in SSc and in mouse models of disease is consistently accompanied by attrition of dermal WAT has kindled great interest in the role of adipocytes and adipose-derived factors in skin fibrosis. Adiponectin, the most abundant circulating adipokine, is decreased in patients with SSc, and shows negative correlation with the extent of skin involvement^22–24^. Adiponectin has pleiotropic actions *in vivo*, and its biology and significance in human fibrosis are poorly understood. We present evidence that adiponectin activity is substantially reduced within the lesional skin in SSc patients. In mice, loss of adiponectin is associated with exaggerated cutaneous fibrosis, while adiponectin overexpression is protective from both skin and peritoneal fibrosis. AdipoR agonist peptides inhibited *ex vivo* fibrotic responses, and prevented and reversed skin fibrosis in mice. These anti-fibrotic effects were accompanied by attenuated FAK activation within lesional tissue. Together, our results implicate, for the first time, deregulated adiponectin expression and function as a key pathogenic mechanism underlying skin fibrosis, and suggest that strategies to augment cellular adiponectin signaling represent novel approaches to SSc therapy.

Skin fibrosis in SSc is mediated by myofibroblasts originating from resident fibroblasts and various mesenchymal progenitor cells. In contrast to the tightly regulated physiological process of wound healing, in fibrosis the endogenous mechanisms that normally restrain myofibroblast activation appear to fail^42^. Accordingly, there is great interest in identifying the regulatory mechanisms and factors that normally prevent excessive fibrogenesis, and characterizing their dysfunction in pathological fibrosis. Skin fibrosis is accompanied by dermal WAT attrition and adipocyte depletion in patients with SSc, as well as in mouse models^9, 43–46^. The adipocyte layer subjacent to the dermis, previously classified as subcutaneous adipose tissue, is increasingly recognized as a distinct adipose depot with unique embryological origins, secretory profiles and physiological functions^4, 5, 45^. Intradermal adipocytes play important roles in skin homeostasis during inflammation, microbial infection, hair cycle, cutaneous aging and wound healing^6–9, 47^. Moreover, dermal WAT acts as an essential thermal insulator and a source of mesenchymal progenitor cells. These homeostatic actions are mediated via adipokines. Adiponectin, secreted by differentiated adipocytes, is a 247-amino acid modular polypeptide found at µg/ml levels in the circulation^18^. Unique among adipokines, adiponectin is most highly expressed in lean adipocytes, and its expression is down-regulated in obesity^48^. Adiponectin elicits pleiotropic actions via ubiquitously expressed AdipoR1, and AdipoR2, which is more restricted to the liver^49^. We recently demonstrated that adiponectin had anti-fibrotic actions mediated via AMP-activated protein kinases^25^. Moreover, RNAi knockdown of endogenous adiponectin resulted in significant fibrotic responses even in the absence of TGF-ß, suggesting a novel cell-autonomous regulatory role for adiponectin.

To examine the relationship between skin fibrosis and altered endogenous anti-fibrotic mechanisms, we evaluated adiponectin signaling in SSc skin biopsies. Using two complementary strategies, we identified a subset of SSc skin biopsies showing significant down-regulation of adiponectin signaling activity associated reduction of AMPK activation. Transgenic mice lacking adiponectin developed exaggerated skin fibrosis, extending on the range of altered biological responses in adiponectin KO mice, including spontaneous pulmonary hypertension and exaggerated cardiac fibrosis^50^. In contrast ΔGLY mutant mice with chronically elevated endogenous adiponectin have been shown to be protected from hyperglycemia, adipose tissue hypoxia, protease inhibitor-induced lipodystrophy, as well as brain inflammation, renal fibrosis and pulmonary hypertension^33, 51–53^, and we found that they were protected from fibrosis in two distinct mouse models. Moreover, ΔGLY-APN mice were also protected from peritoneal fibrosis. Together, these studies provide strong evidence that endogenous adiponectin exerts powerful negative regulatory effects on fibrosis, and suggest that impaired adiponectin signaling may contribute to persistence of skin fibrosis in SSc. ADP355 and related peptides with AdipoR1/2 receptor agonist activity show efficacy in models of cancer, lipodystrophy and brain inflammation^37^. In the present studies chronic ADP355 treatment was well tolerated and resulted in attenuation of skin fibrosis, and were associated with AMPK activation and blockade of FAK activation. In light of the recognized importance of FAK activation and focal adhesion complex assembly in skin and lung fibrosis^34^, targeting FAK activity represents a novel approach to blocking fibrotic responses while preserving physiological TGF-ß signaling.

In summary, we demonstrate that SSc skin fibrosis is associated with impaired adiponectin signaling within lesional tissues. In mice, loss of endogenous adiponectin is associated with increased sensitivity to skin fibrosis, whereas physiologic-range modest elevation of adiponectin affords substantial protection from both skin and peritoneal fibrosis. Moreover, targeting cellular adiponectin receptors with synthetic agonist peptides elicits potent anti-fibrotic effects in normal and SSc skin fibroblasts *in vitro*, and prevents and reverses skin fibrosis in mice. These observations provide support for a homeostatic role for adiponectin in fibrosis, and implicate loss of this activity in persistence of fibrosis in SSc. In light of their tolerability and favorable pharmacokinetic properties, peptides activating adiponectin pathways might represent viable tools for further development as anti-fibrotic therapies.

## Experimental Procedures or Methods

### Human subjects

Subjects with SSc (n = 19) and healthy volunteers (n = 4) were recruited from the Northwestern Scleroderma Clinics (Supplementary Table 1). All patients fulfilled American College of Rheumatology (ACR) criteria for the classification of SSc^54^. Information obtained at the time of tissue collection included demographics, disease duration (defined as the interval between the first non-Raynaud SSc manifestation and sampling) and modified Rodnan skin score (MRSS, range 0–51). Patients were grouped into early- (<24 months) or late- (>24 months) stage subsets. Written informed consent was obtained from all participants. The protocol of this study was approved by Northwestern University Institutional Review Board for Human Studies, and was in accordance with the Helsinki Declaration as revised in 2000.

### Animals

C57BL6/J female mice (8–12 wk old) (Jackson Laboratories, Bar Harbor, ME, USA, #00664), and ΔGLY-APN transgenic mice^33, 53^ and adiponectin knockout (APN KO) mice^56^, both in the isogenic C57BL6 background, were housed at constant temperature on a 12 h light/dark cycle, and given regular chow and water ad libitum. All mice were genotyped using genomic DNA isolated from tail biopsies. Experimental procedures complied with the Public Health Service Policy on Humane Care and Use of Laboratory Animals and all animal protocols were approved by the Institutional Animal Care and Use Committee of Northwestern University or Tokyo University.

### Induction of fibrosis

Bleomycin (BLM) or phosphate buffered saline (PBS) was given by daily subcutaneous (s.c.) injection for 14 consecutive days^55^. In some experiments, mice were given a single s.c. injection of adenovirus expressing constitutively-active TGF-ß1 (TGFß1^223/225^ 3x10^9^ PFU/ml) or beta-galactosidase (5x10^9^ PFU/ml)^56^. Peritoneal fibrosis was induced by i.p. injections of 0.1% chlorhexidine gluconate (CG; Wako Pure Chemical Industries, Osaka, Japan) dissolved in 15% ethanol/PBS given on alternate days for 21 days^35^. Synthetic peptides targeting AdipoR (ADP355 or ADP27)^37^ or vehicle (PBS) at the indicated concentrations were administered by daily i.p. injection for 14 or 24 days. Each experimental group consisted of 4–8 mice, and experiments were repeated at least two times with consistent results. At the end of the experiments, mice were sacrificed and blood and tissue were collected for analysis^12^.

### Synthesis and Purification of AdipoR Agonist Peptides

The peptides ADP355 (H-DAsn-Ile-Pro-Nva-Leu-Tyr-DSer-Phe-Ala-DSer-NH2) and ADP27 (H-Leu-Tyr-Tyr-Phe-Ala-Tyr-His-Ile-Thr-Val-NH2) were synthesized in solid-phase using a CEM Liberty microwave assisted peptide synthesizer utilizing Fmoc-chemistry, RP-HPLC purification, and MALDI-MS verification^37^. Following purification, peptides were lyophilized twice from 2% aqueous acetic acid solution to switch to acetate form.

### Peptide binding and *in vivo* biodistribution

To evaluate *in vitro* peptide binding, skin fibroblasts seeded on coverslips were preincubated with unlabeled ADP355 (100 μg/ml) for 10 min, followed by labeled ADP355-Dy675 (5 μg/ml), or labeled control peptide (Api88 Dy675, gift from Daniel Knappe, Leipzig, Germany) for 30 min. Cells were fixed in 4% phosphate buffered paraformaldehyde (PFA) and visualized using a Nikon A1 confocal microscope^57^. To evaluate *in vivo* biodistribution, C57BL6/J mice were injected i.p. with Dy675-coupled peptide (20 μg/mouse or 200 μg/μl) and 10 min later, dorsal skin was harvested and processed for immunofluorescence analysis.

### Evaluation of fibrosis

Harvested skin or parietal peritoneal membrane samples were fixed in PFA, embedded in paraffin and 4 μm thick sections were stained with hematoxylin and eosin (H&E). Collagen deposition and organization were assessed using Masson’s Trichrome staining. Thickness of the dermis, dWAT or peritoneal membrane were determined at five randomly selected locations/slide using ImageJ software^9^.

### Immunofluorescence

Four μm thick paraffin-embedded sections of lesional skin or peritoneal membrane were incubated with rabbit anti-phospho-AMPK (Thr172) (1:100, Cell Signaling Technology, Beverly, MA), mouse anti-α-SMA (1:250, Dako, Carpinteria, CA), rabbit anti-α-SMA (1:200, Abcam, Cambridge, UK), rat anti-procollagen I (1:200, EMD Millipore, Billerica, MA), goat anti-type III collagen (1:200, Southern Biotech, Birmingham, AL), rabbit anti-Ki67 (1:500, Abcam) or rabbit anti-phospho FAK (Y397; 1:50, Abcam) primary antibodies, followed by species-appropriate secondary antibodies conjugated to Alexa Fluor 488, 594 or 647 (Invitrogen, Carlsbad, CA). Nuclei were detected using 4′,6-diamidino-2-phenylindole (DAPI). Slides were evaluated using a Nikon A1 laser scanning confocal microscope.

### Immunocytochemistry

Normal skin fibroblasts (10,000 cells/well) seeded in 8-well Lab-Tek II chamber glass slides (Nalge Nunc International, Naperville, IL) were incubated in media with indicated reagents. At the end of the experiments, cells were fixed, permeabilized, and incubated with primary antibodies to α-SMA (1:500, Sigma), phospho-FAK (pY397; 1:50, Abcam), vinculin (1:200, Abcam), Alexa Fluor 488 phalloidin (1:200, Invitrogen) or Type I collagen (1:100, Southern Biotech), followed by appropriate secondary antibodies conjugated to Alexa Fluor (Invitrogen), and evaluated under a Nikon C1Si confocal microscope^58^.

### Measurement of collagen and adiponectin

Collagen content of lesional skin was determined by measuring the hydroxyproline content in 6-mm punch biopsy samples (BioVision, Milpitas, CA) (16). Serum levels of adiponectin were determined by ELISA which measures all adiponectin isoforms (Millipore; #EZMADP-60K)^9^.

### Cell cultures and reagents

Primary cultures of healthy adult and SSc skin fibroblasts, and neonatal fibroblasts, were established by explantation^25^. The clinical features of the subjects are shown in Supplementary Table 5. Protocols for skin biopsies were approved by the Institutional Review Board at Northwestern University. Embryonic fibroblasts from AMP protein kinase (AMPK)^−/−^ mice and wild-type mice were a gift from Yu-Ying He (University of Chicago). When cultures reached confluence, serum-free media supplemented with 0.1% bovine serum albumin (BSA) were added prior to TGF-β2 (Peprotech, Rocky Hill, NJ) or/and ADP355 (10 μM)^37^, and incubation continued for a further 24 h. Cytotoxicity was evaluated using LDH assays (Biovision).

### Three-dimensional human skin equivalents

Normal human skin fibroblasts (3 × 10^5^cells) were mixed with rat tail Type I collagen (4 mg/ml, BD Biosciences, San Jose, CA) and seeded in 12-well plates^59, 60^. Epidermal keratinocytes (6 × 10^6^ cells) were seeded on the collagen plugs. Forty-eight hours later, the 3D organotypic cultures were placed on metal grids (BD Biosciences) and maintained for 5 days at an air-medium interface^25^. Peptides (10 uM) were then added in media with or without TGF-β, and incubations continued for a further six days. Experiments were harvested, and RNA was isolated for analysis.

### Transient transfection assays

Fibroblasts at early confluence were transfected with [SBE]_4_-luc plasmids or indicated reporter constructs using SuperFect Transfection (Qiagen)^61^. Cultures were incubated in serum-free media containing 0.1% BSA for 16 h, followed by ADP355 and TGF-β for a further 24 h^61^. Whole cell lysates were assayed for luciferase activities using the dual-luciferase reporter assay system (Promega, Madison, WI). In each experiment, Renilla luciferase pRL-TK (Promega) was cotransfected as control for transfection efficiency^62^. Experiments were performed in triplicate and repeated at least twice with consistent results. For RNAi-mediated transcript silencing, fibroblasts were transfected with target-specific short interfering siRNA specific for AdipoR1/R2 (Dharmacon, Lafayette, CO) or scrambled control siRNA (10 nM, unless otherwise indicated). Twenty-four hours following transfection, fresh media containing TGF-β (2 ng/ml) or ADP355 (10 μM) were added to the cultures and incubation continued for a further 24 h.

### Quantitative real-time PCR (qPCR)

RNA was isolated from skin samples using RNAeasy Fibrous Tissue Mini kit (Qiagen, Valencia, CA), or from fibroblast cultures, using the RNeasy Plus mini kit (Qiagen), and processed for qPCR as described^9^. mRNA levels were normalized to the levels of 18 S RNA or GAPDH, and the relative amounts were calculated using the 2^−ΔΔCt^ method^63^.

### Western analysis

At the end of the experiments, fibroblasts were harvested and whole cell lysates (100 μg) were subjected to Western-blot analysis^25^ using antibodies for Type I collagen (Southern Biotech), α-SMA (Sigma), phospho-Smad2/3 (Cell-Signaling), phospho-FAK (pY397, Cell Signaling), total-FAK (Cell Signaling) or GAPDH (Zymed, San Francisco, CA). Electrophoretic bands were detected using enhanced chemiluminescence reagents (Pierce Biotechnology, Rockford, IL), and band intensities were quantified with Image J software. Results were normalized with GAPDH levels in each sample.

### Derivation and measurement of adiponectin pathway scores in skin biopsies

To interrogate the expression of adiponectin-regulated genes and assess adiponectin pathway activation, we selected the top 20 genes up-regulated and down-regulated by adiponectin from a publicly available microarray dataset (GSE49332). Those genes also present on Agilent human arrays (16 up-regulated and 13 down-regulated) were extracted from a publicly available microarray dataset (GSE76886) of skin biopsies from SSc patients (n = 70; prior to immunomodulatory treatment) and control (n = 22) subjects.

Adiponectin signature scores were calculated based on published approaches^25^. The mean and SD levels of each adiponectin-regulated gene in the healthy control group was used to standardize expression levels of each gene for each skin biopsy. The standardized expression levels were subsequently summed for each biopsy to provide an adiponectin signature score based on the following formula:

Σ^*n*^_*i=1*_ = (*GENEiSSc* − MEANictr/SDctr) *k, where i = each of the adiponectin-regulated genes, GENEi_SSc_ = gene expression level in each SSc biopsy, and MEANi_ctr_ = average gene expression in controls. For adiponectin-induced genes, k = 1; for adiponectin-suppressed genes, k = −1. To further assess the network of genes associated with adiponectin expression, levels for all genes queried in the GSE76886 were correlated to adiponectin expression using Pearson correlation coefficient. The top 500 most differentially expressed (up- or down-regulated) transcripts were then used to generate a heatmap comparing SSc patients and healthy controls. The entire rank-ordered gene list was then input into the Gene Ontology enRIchment anaLysis and visuaLizAtion tool (GOrilla) database for further analysis of gene ontology^64^.

### Statistical analysis

Results are presented as the mean ± SD. For group comparisons, two-sample *t*-test, Wilcoxon-Mann-Whitney test, or Analysis of Variance (Bonferroni correction for multiple comparisons) were used. *P* values less than 0.05 were considered significant. Statistical analyses were performed with GraphPad Prism version 6.00 for Windows (GraphPad Software, La Jolla California USA, www.graphpad.com).

## Electronic supplementary material

Supplementary information
