# Supplementary material for: Adiponectin is an endogenous anti-fibrotic mediator and therapeutic target
Source: Sci Rep. 2017 Jun 30;7:4397. doi: 10.1038/s41598-017-04162-1 (PMC5493638; doi:10.1038/s41598-017-04162-1)
Supplement: Supplementary file 1 — Supplementary information [file 41598_2017_4162_MOESM1_ESM.pdf]

SUPPLEMENTARY INFORMATION

Adiponectin is an endogenous anti-fibrotic mediator and therapeutic target

Roberta G Marangoni[1], Yuri Masui[2], Feng Fang[1], Benjamin Korman[1], Gabriel Lord[1], Junghwa Lee[3], Katja Lakota[4], Jun Wei[1], Philipp E. Scherer[5], Laszlo Otvos Jr.[6], Toshimasa Yamauchi[7], Naoto Kubota[7], Takashi Kadowaki[7], Yoshihide Asano[2], Shinichi Sato[2], Warren G. Tourtellotte[8], John Varga[1]*

SUPPLEMENTARY FIGURE LEGENDS

Supplementary Fig. 1. Circulating adiponectin levels correlate negatively correlate with extent of skin fibrosis.

Transgenic (ΔGLY-APN) and wildtype (WT) mice received daily s.c. injections of bleomycin (BLM) or PBS (control) for 14 days. Serum and lesional skin were collected at day 21. **(a)** Serum adiponectin was measured by ELISA; results are means of duplicate determinations. Horizontal bars indicate the mean ± SD from three independent experiments. Mann–Whitney U-test. **(b)** Correlation between adiponectin levels and dermal thickness (left panel) or skin hydroxyproline (right panel) levels in bleomycin-treated mice. Spearman's rank correlation test.

Supplementary Fig. 2. Reduced collagen stimulation in ΔGLY-APN transgenic mice

ΔGLY-APN and wildtype (WT) mice received daily s.c. injections of bleomycin (BLM) or PBS (control) for 4 d, and skin was harvested at day 5. Immunofluorescence using antibodies to procollagen I (red). Representative photomicrographs. Scale bars = 50 μm. Dotted lines indicate epidermal/dermal junction. Insets, procollagen I-positive cells within dermis, scale bar = 10 μm. **(b)** Immunopositive cells were counted. Results are means ± SD from three hpf/slide.

Supplementary Fig. 3. Adiponectin attenuates focal adhesion assembly and focal adhesion kinase activation.

ΔGLY-APN and wildtype (WT) mice received daily s.c. injections of bleomycin (BLM) or PBS (control) for 14 d. Lesional skin was examined by immunofluorescence at day 21

using antibodies to phospho-FAK (Y397) and αSMA. Representative photomicrographs; scale bar, 20 µm. Immunofluorescence intensity of p-FAK on αSMA-positive myofibroblasts was determined. Results, corrected for total cell fluorescence, shown as means ± SD.

Supplementary Fig. 4. ΔGLY-APN transgenic mice are protected from TGF-ß1-induced skin fibrosis.

ΔGLY-APN and wildtype (WT) mice received a single s.c. injection of Ad-TGFß1 or Ad-LacZ (control), and lesional skin was harvested 42 d later. L. **(a)** Masson's Trichrome stain. Representative images; scale bar = 100 µm. **(b)** Dermal thickness. Results are means ± SD of 5 determinations/mouse (5 mice/group). **(c)** Herovici stain. Mature (pink) and immature (blue) collagen fibers. Representative images; scale bar = 50 µm. **(d)** Real-time qPCR. Results are means ± SD from four mice/group.

Supplementary Fig. 5. ΔGLY-APN transgenic mice are protected from peritoneal fibrosis

Transgenic (ΔGLY-APN) and wildtype (WT) mice received alternate daily i.p. injections of chlorhexidine gluconate (CG) or vehicle (control) for indicated periods. Mice were sacrificed and peritoneal membranes harvested for analysis. **(a)** Masson's Trichrome stain. Representative images; scale bar = 50 µm. Right panel, peritoneal membrane thickness. Results are the mean ± SD of five determinations/mouse (4 mice/group). **(b,c)** Immunohistochemistry using antibodies to (B) Ki67 or (C) αSMA; scale bars = 50 µm. Immunopositive cells were quantified. Results are mean ± SD.

Supplementary Fig. 6. Labeled ADP355 is distributed throughout the skin *in vivo* and binds specifically to fibroblasts *in vitro*

(a) Neonatal skin fibroblasts were incubated *with ADP355 for 30 min*, followed by control peptide (Api88-Dy675), ADP355-Dy675 or unlabeled ADP355. Representative photomicrographs from three independent experiments. **(b)** C57BL6/J mice received a single i.p. injection of ADP355-Dy675 or control peptide (Api88-Dy675). Dorsal skin was harvested 10 min later for immunofluorescence. Photomicrographs representative of two independent experiments (3 mice per group).

Supplementary Fig 7. ADP355 attenuates Smad2/3 activation

Fibroblasts preincubated with ADP355 were incubated with TGF-β for 24 h. Whole cell lysates were examined by Western blot analysis. Band intensities of Smad2 normalized to total Smad2/3 levels in each lane are shown.

Supplementary Fig. 8. ADP355 attenuates focal adhesion assembly and focal adhesion kinase activation

(a-c). Confluent dermal fibroblasts were pre-incubated in media with ADP355, followed by TGF-β2 for 24 h or indicated periods. **(a)** Whole cell lysates were examined by Western analysis. Representative immunoblots. -Fold change in p-FAK (Y397) levels normalized to total FAK. **(b,c)** Fibroblasts were immunostained with antibodies to F-actin (green) and pFAK (Y397) (red); or vinculin (green) and F-actin (red); or stained

with DAPI. Representative immunofluorescence photomicrographs. Original magnification x 400.

Supplementary Fig. 9. ADP27 prevents and reverses bleomycin-induced skin fibrosis

C57BL6/J mice received bleomycin (BLM) or PBS via daily s.c. injections. Daily i.p. injections of ADP27 (0.2 ug/ul, 100 ul in PBS) were initiated concomitantly with, or starting on day 7, of BLM. Mice were sacrificed on day 24 and lesional skin harvested for analysis. **(a)** Masson's trichrome stain. Representative images; scale bar = 100 µm. **(b)** Dermal thickness. Results are means ± SD of five determinations per mouse. **(c)** Gastrocnemius muscles were harvested 10 min following final ADP27 injection, and lysates examined by Western analysis. Representative autoradiographs. -Fold change in levels of phospho-AMPK (p-AMPK, Thr172) relative to total AMPK is shown.

Supplementary Figure 1

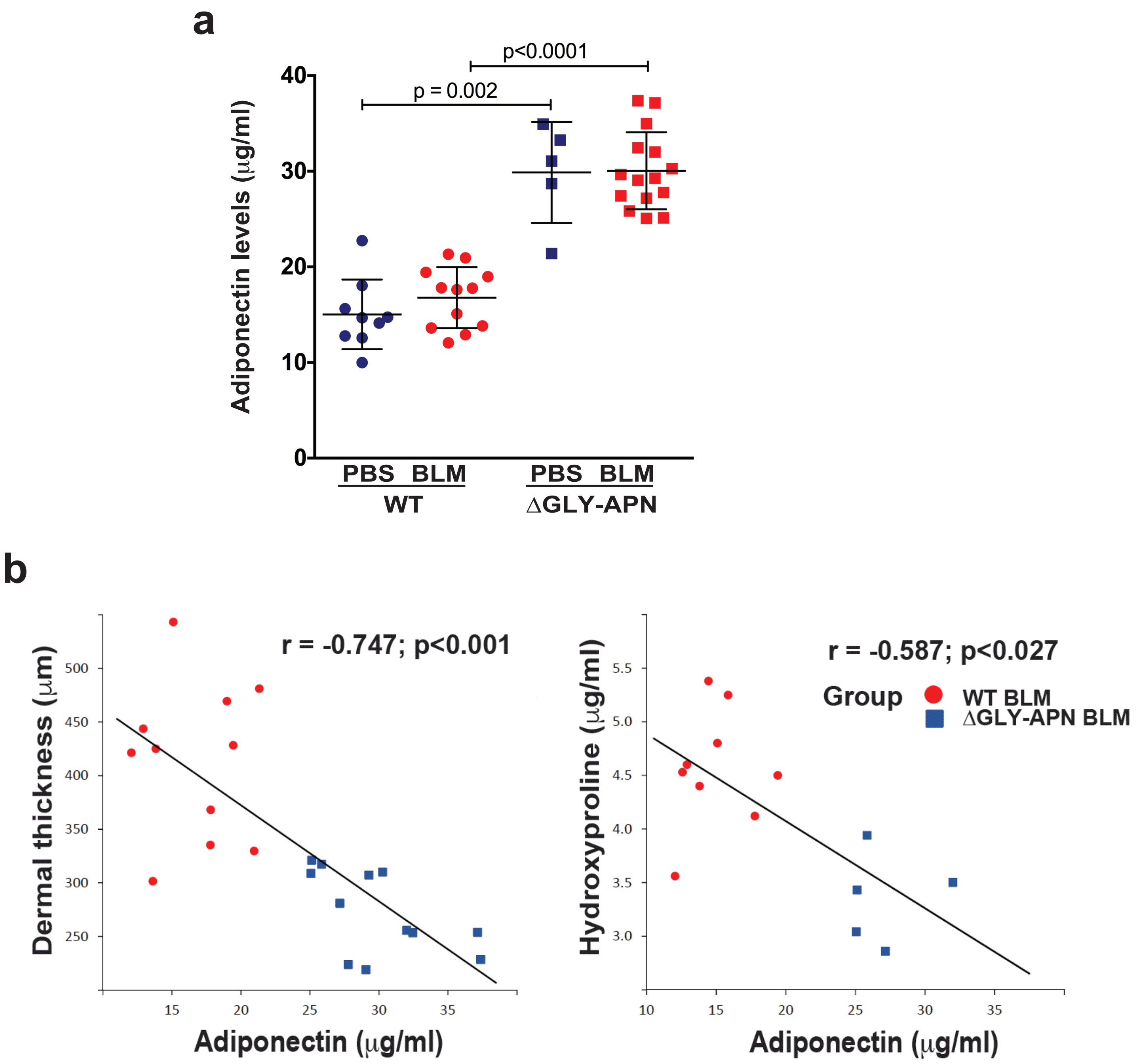

Supplementary Figure 2

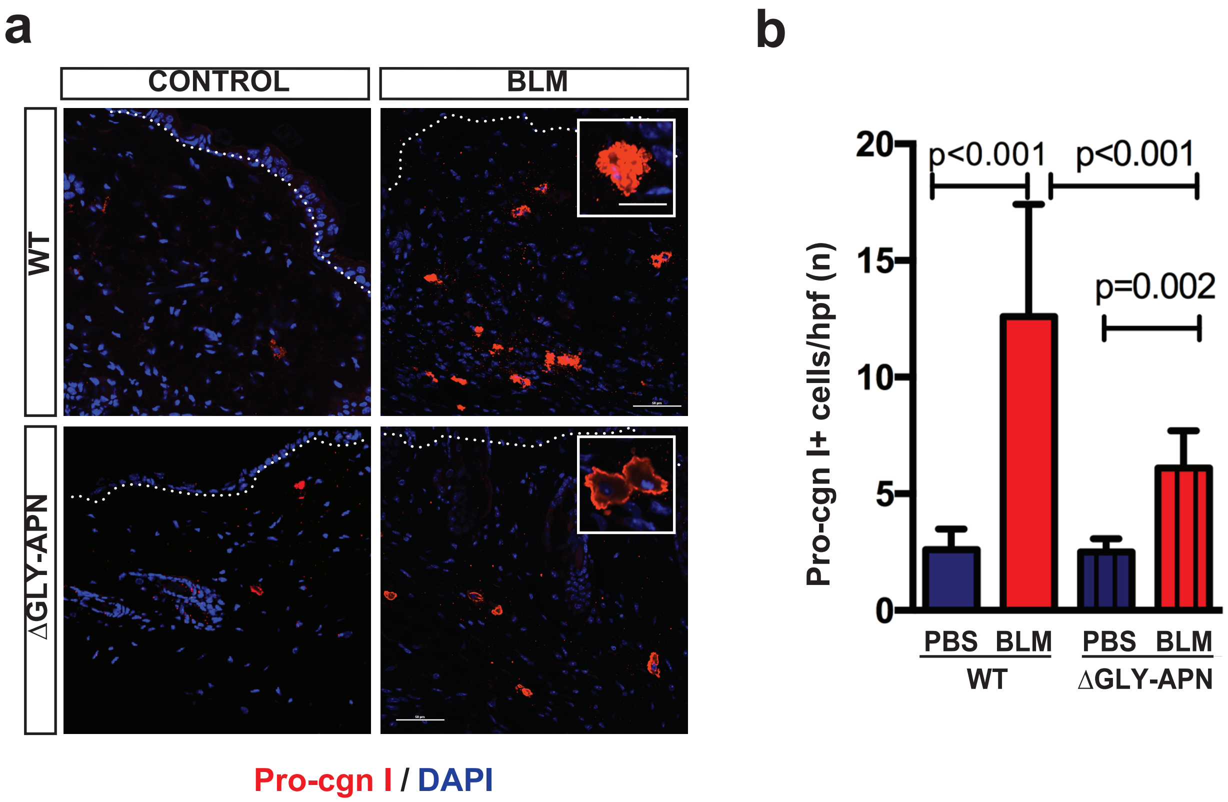

Supplementary Figure 3

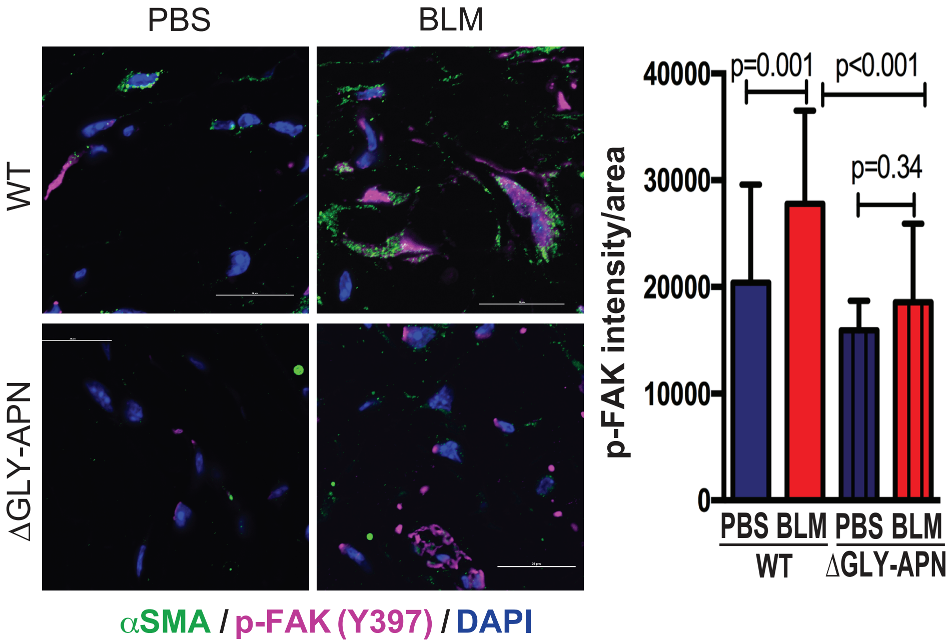

Supplementary Figure 4

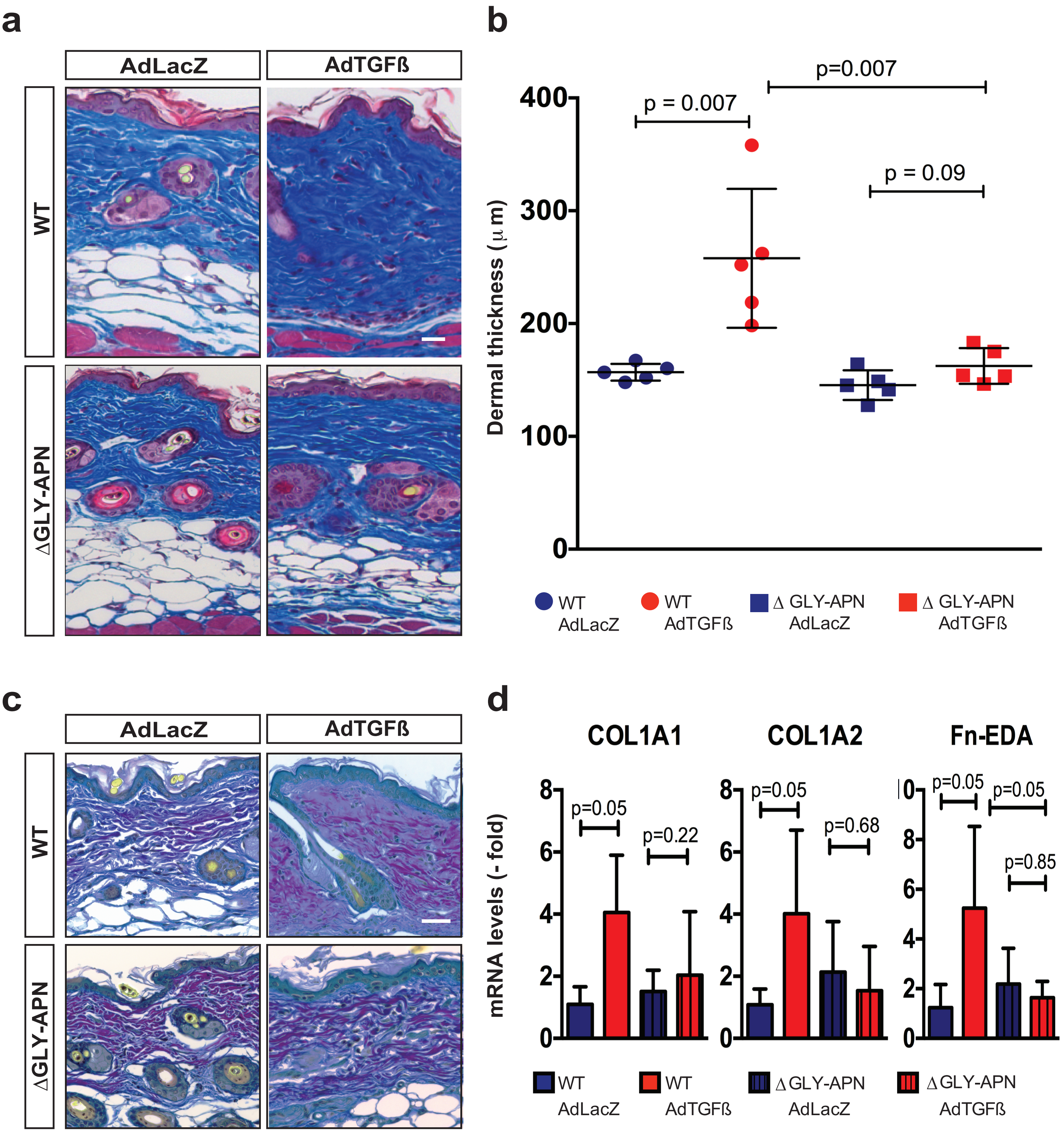

Supplementary Figure 5

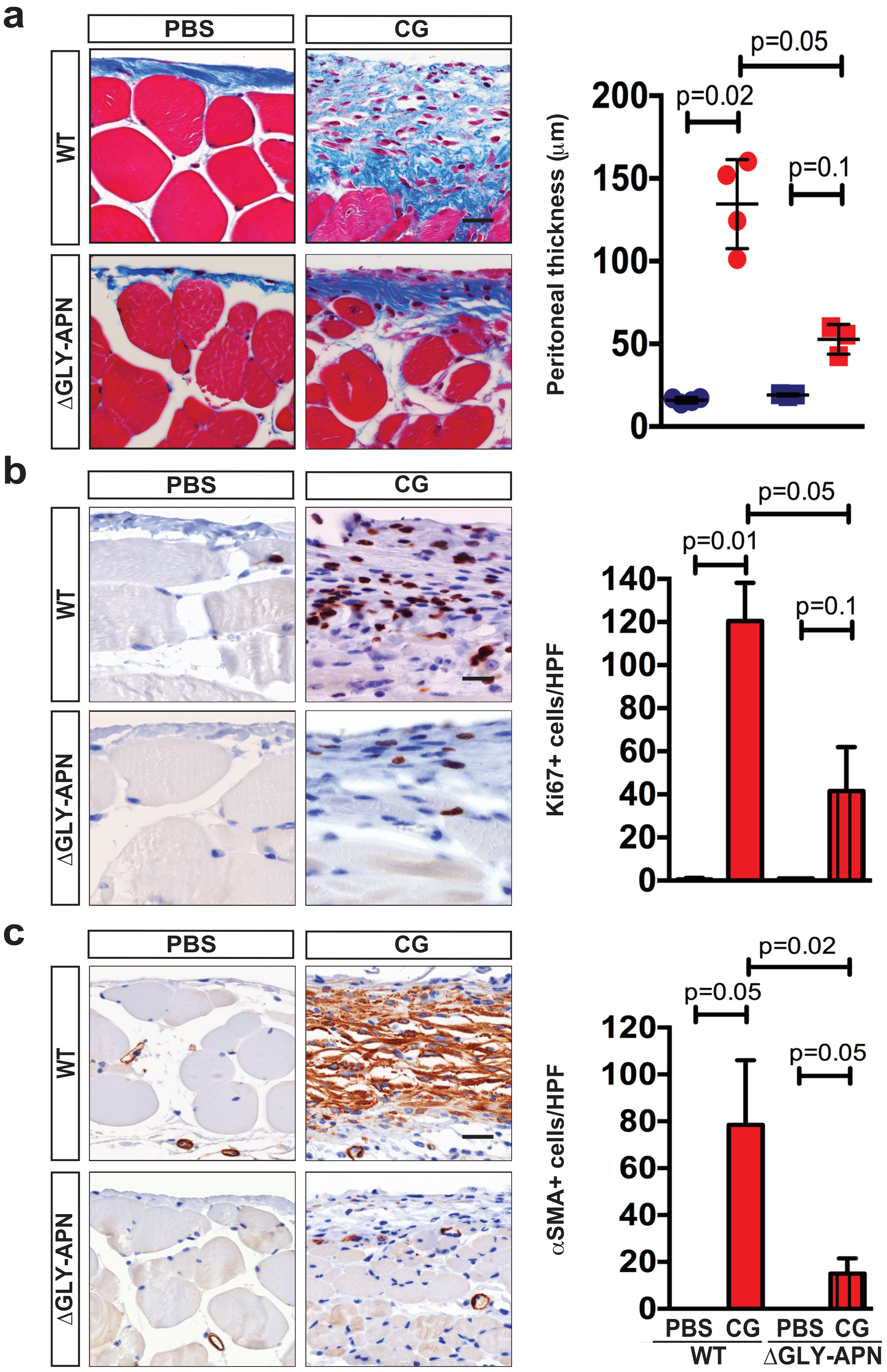

Supplementary Figure 6

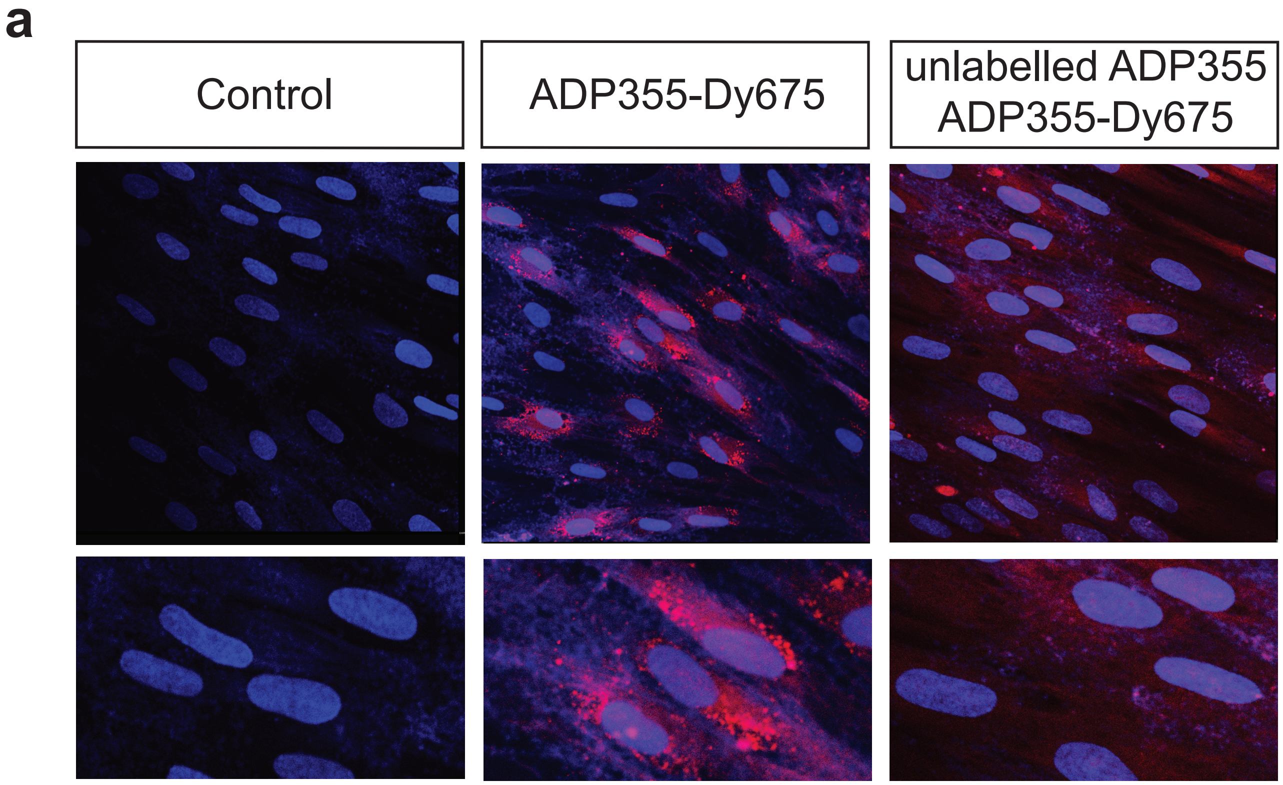

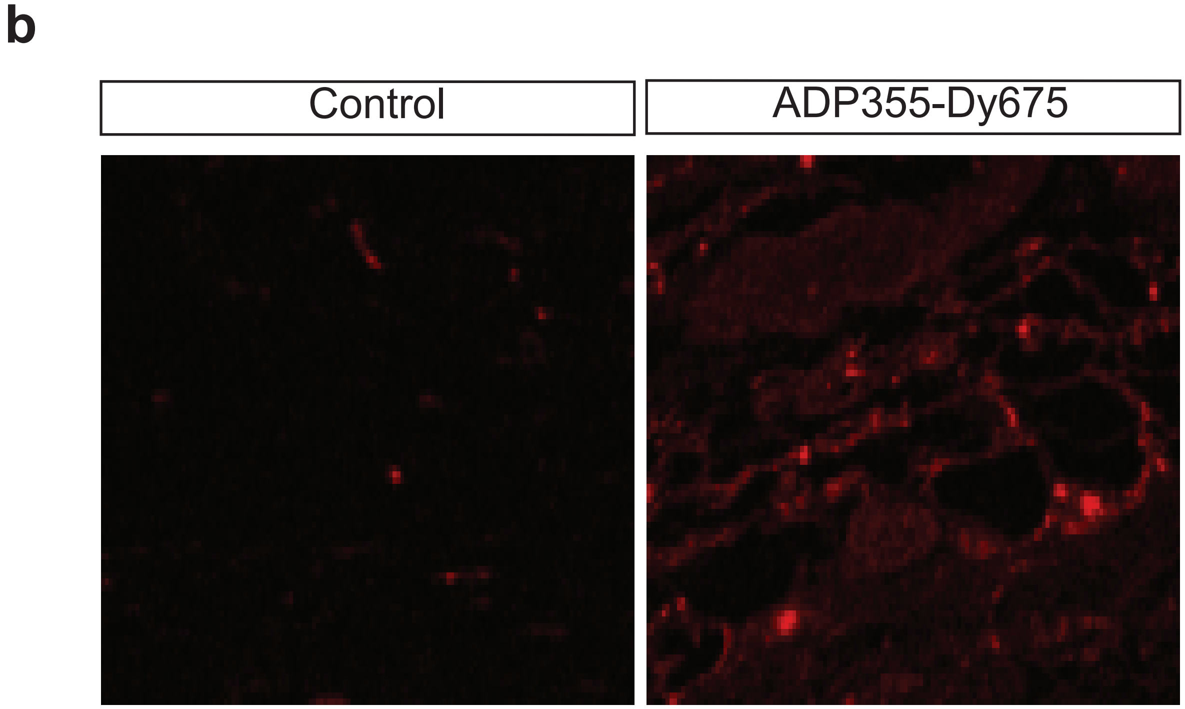

Supplementary Figure 7

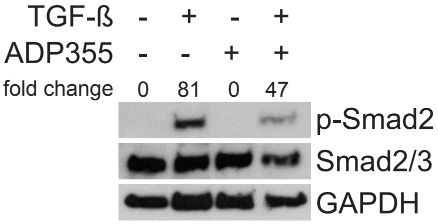

Supplementary Figure 8

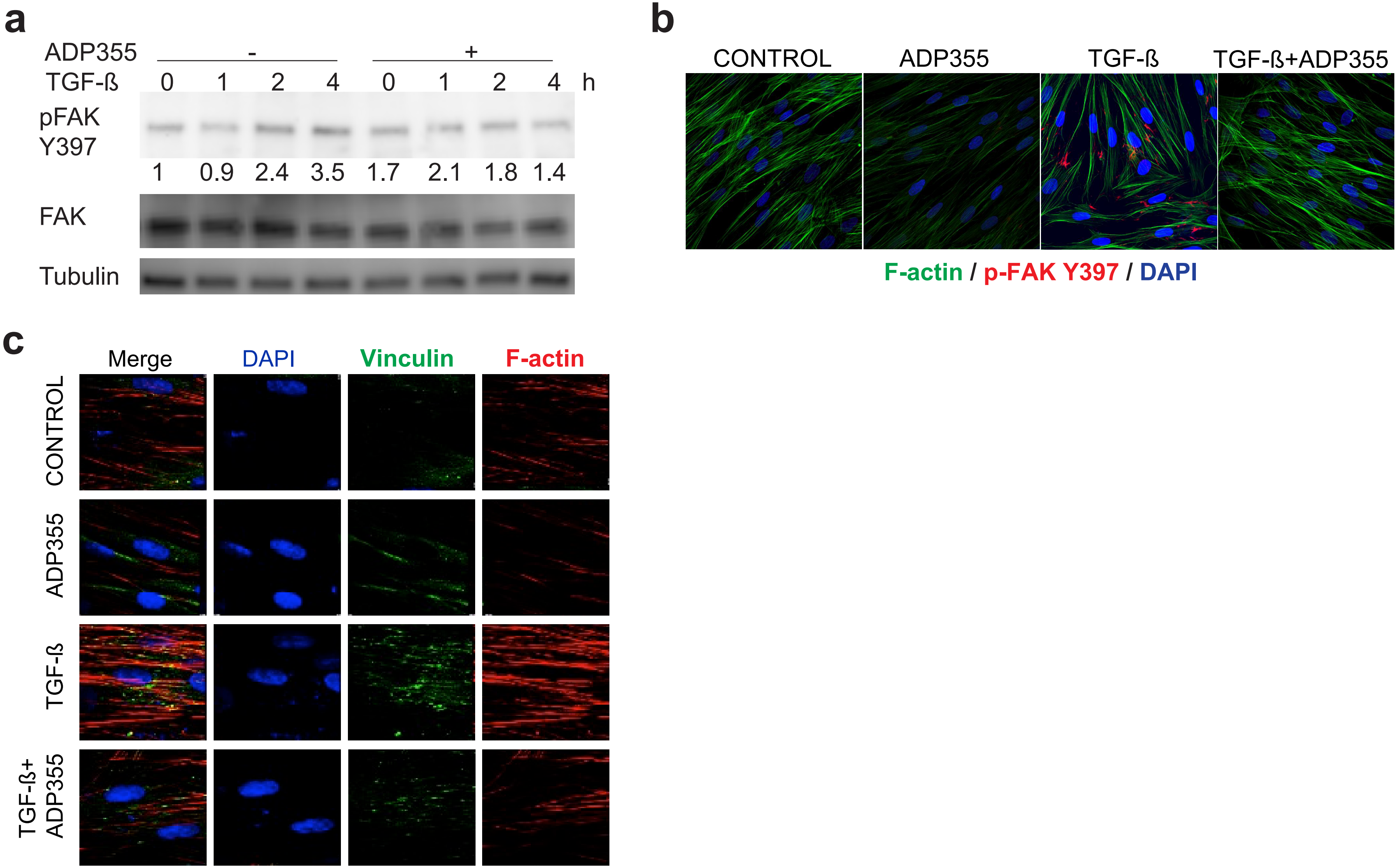

Supplementary Figure 9

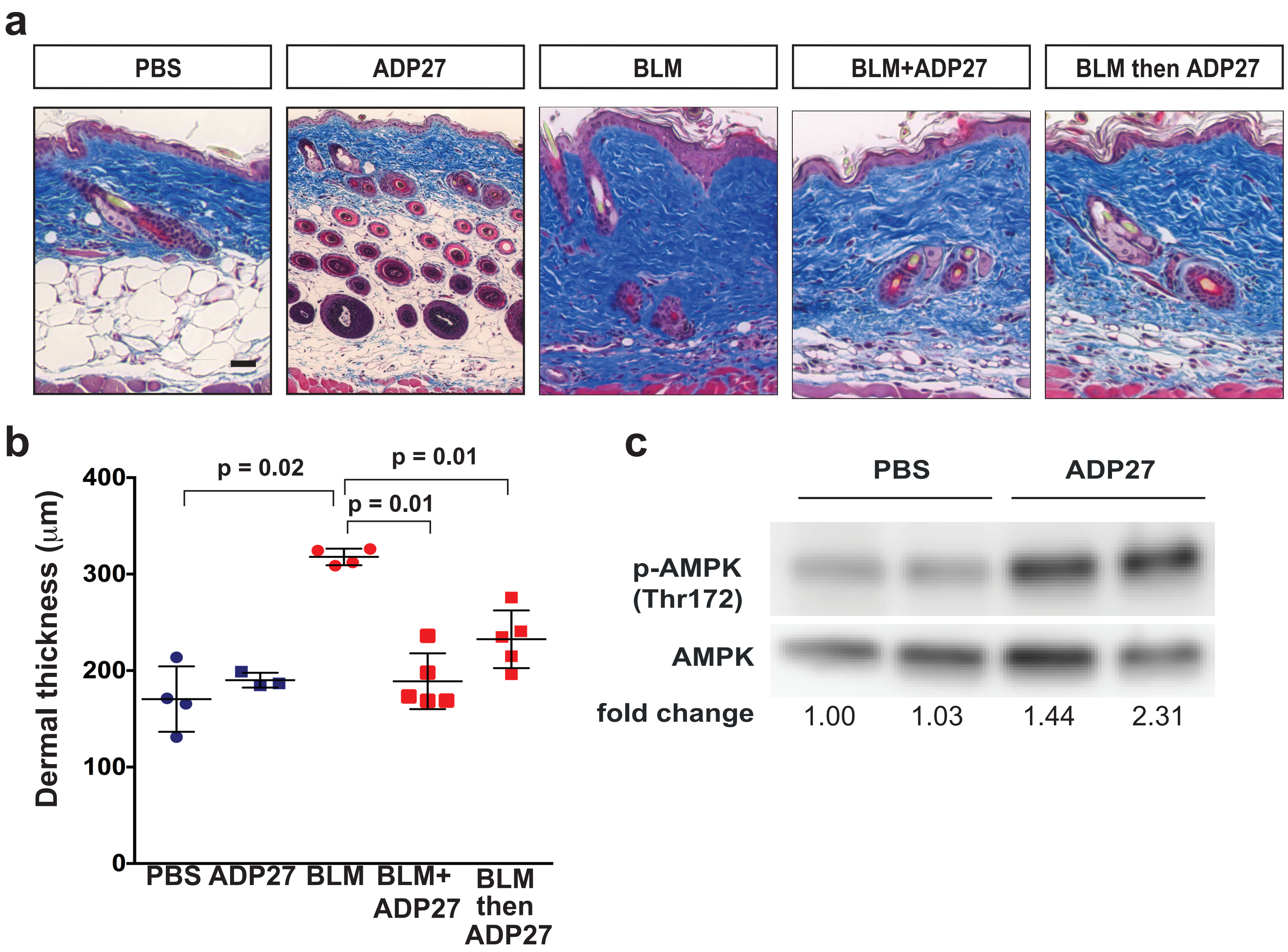

Table 1. Demographic and clinical characteristics of SSc patients and healthy controls providing skin biopsies for immunofluorescence analysis.

Study Code	Age (yrs)	Sex	Onset	Subtype	MRSS
RegNorm1019	44	F			
RegNorm1020	34	F			
MHNorm14	34	F			
MHNorm16	37	F			
SScReg1207	65	F	Late	lcSSc	3
SScMH07	62	F	Late	lcSSc	3
SScMH32	30	F	Late	lcSSc	1
SScMH35	56	F	Late	lcSSc	6
SScMH41_6mo	56	F	Late	lcSSc	3
SScMH23_24mo	57	F	Late	lcSSc	4
SScMH36_6mo	19	F	Early	lcSSc	0
SScMH08_12mo	67	F	Early	dcSSc	10
SScMH42_6mo	48	M	Early	dcSSc	10
SScMH21_6mo	44	F	Early	dcSSc	7
SScReg1523_Base	39	M	Early	dcSSc	13
SScReg1402_Base	50	F	Early	dcSSc	14
SScMH12_6mo	52	F	Early	dcSSc	17
SScMH10_6mo	53	F	Early	dcSSc	13
SScMH55_6mo	51	F	Early	dcSSc	35
SScMH17_24mo	55	M	Late	dcSSc	19
SScMH01_6mo	52	F	Late	dcSSc	24
SScReg1582_Base	31	F	Late	dcSSc	25
SScMH20_6mo	60	F	Late	dcSSc	16

Subjects providing skin biopsy for histology. dcSSc, diffuse cutaneous SSc; lcSSc, limited cutaneous SSc; M, male, F, female. MRSS, modified Rodnan skin score (1-51). Controls were healthy subjects. Early onset, <2 years from first non Raynaud's symptom.

Table 2. List of genes used to define a synexpression gene set

Agilent ID	Gene name	Correlation with adiponectin
A_23_P369237	adiponectin, C1Q and collagen domain containing	1
A_23_P204736	glycerol-3-phosphate dehydrogenase 1 (soluble)	0.788996051
A_24_P397817	leptin	0.726015528
A_23_P27846	KIAA1881	0.71506561
A_23_P376704	cell death-inducing DFFA-like effector a	0.691648918
A_23_P72697	glycosylphosphatidylinositol anchored high density lipoprotein binding protein 1	0.688732589
A_23_P26154	perilipin	0.686632602
A_23_P39056	kallikrein-related peptidase 7	0.682302221
A_23_P58359	alcohol dehydrogenase 1B (class I), beta polypeptide; alcohol dehydrogenase	0.677718747
A_32_P437004	Xg blood group	0.658955175
A_23_P110319	hypothetical protein FLJ21511	0.654742787
A_23_P164504	desmoglein 1	0.64932792
A_24_P206776	crystallin, alpha B	0.647379634
A_23_P247		0.646363895
A_24_P209455	GTPase, IMAP family member 4	0.644580626
A_24_P913146	HOP homeobox	0.640753123
A_23_P103765	Fc fragment of IgE, high affinity I, receptor for;	0.640664966
A_23_P140454		0.640418396
A_23_P123853	chemokine (C-C motif) ligand 19	0.63743974
A_23_P325690	ankyrin repeat domain 35	0.636570426
A_23_P65388	C-type lectin domain family 14, member A	0.633650337
A_23_P42969	fibrinogen-like 2	0.633439805
A_24_P269527	secreted LY6/PLAUR domain containing 1	0.633239831
A_23_P81158	alcohol dehydrogenase 1B (class I), beta polypeptide; alcohol dehydrogenase	0.633206363
A_23_P218669	WAP four-disulfide core domain 5	0.632835763
A_23_P26386	tubulin polymerization-promoting protein family member 3	0.630887416
A_23_P67661	cytochrome c oxidase subunit VIIa polypeptide 1 (muscle)	0.629935642
A_23_P114597	small proline-rich protein 2C (pseudogene); small proline-rich protein 2G	0.628260108
A_23_P101380	UDP-GlcNAc:betaGal beta-1,3-N-acetylglucosaminyltransferase 8	0.627981822
A_23_P128855	solute carrier family 39 (zinc transporter), member 2	0.626395389
A_23_P394304	PDZK1 interacting protein 1	0.622886509
A_23_P217269	V-set and immunoglobulin domain containing 4	0.622234381

A_23_P500000	sciellin	0.621624452
A_23_P200741	dermatopontin	0.615106864
A_24_P938284	Fc fragment of IgG, low affinity IIb, receptor (CD32);	0.611572616
A_24_P34199	filaggrin	0.609950555
A_23_P153480	kallikrein-related peptidase 5	0.609733967
A_24_P370472	major histocompatibility complex, class II, DR beta 4; major	0.608868671
A_24_P402222	major histocompatibility complex, class II, DR beta 3	0.60852351
A_23_P105562	von Willebrand factor	0.602124126
A_23_P26965	chemokine (C-C motif) ligand 13	0.60085274
A_24_P51322	filaggrin	0.599419863
A_32_P387648	filaggrin	0.597840476
A_23_P413721	glycerol-3-phosphate dehydrogenase 1 (soluble)	0.593533291
A_23_P157879	ficolin (collagen/fibrinogen domain containing) 1	0.591095793
A_24_P159227	p21 protein (Cdc42/Rac)-activated kinase 6	0.58688743
A_23_P381261	adenylate cyclase 4	0.586715834
A_32_P141948		0.586435503
A_23_P349463	calcineurin B homologous protein 2	0.585257809
A_23_P144911	EGF-like, fibronectin type III and laminin G domains	0.584754997
A_23_P18017	carboxypeptidase A3 (mast cell)	0.580020188
A_23_P170233	cystatin A (stefin A)	0.578485147
A_23_P137366	complement component 1, q subcomponent, B chain	0.577394846
A_23_P24077	chromosome 10 open reading frame 54	0.576966244
A_23_P3312	immunoglobulin superfamily containing leucine-rich repeat	0.57502159
A_23_P11787	wingless-type MMTV integration site family, member 4	0.574511685
A_23_P47709	folate receptor 2 (fetal)	0.572685408
A_23_P138706	adrenergic, alpha-2A-, receptor	0.571448161
A_23_P203558	hemoglobin, beta	0.569784672
A_23_P165598	death associated protein-like 1	0.569313452
A_23_P22013	R-spondin homolog (Xenopus laevis)	0.569080453
A_23_P259561		0.569002194
A_24_P845223		0.565156837
A_24_P892472	EMX2 opposite strand (non-protein coding)	0.562191578
A_23_P6818	sema domain, immunoglobulin domain (Ig), short basic domain, secreted,	0.561871534
A_23_P897	chromosome 1 open reading frame 116	0.560680455
A_24_P170136		0.560212445
A_23_P8083	lymphocyte antigen 6 complex, locus G6C	0.558986852

A_32_P131608		0.558199274
A_23_P52323	collagen, type XVII, alpha 1	0.556780694
A_23_P397248	chloride channel accessory 2	0.555072595
A_23_P162660	glycerol-3-phosphate dehydrogenase 1 (soluble)	0.553477334
A_24_P103469	leukocyte specific transcript 1	0.5532962
A_23_P101992	macrophage receptor with collagenous structure	0.55193335
A_23_P12155	cysteine-rich C-terminal 1	0.548634849
A_23_P134764	lymphocyte antigen 6 complex, locus D	0.547796412
A_23_P37088	retinol dehydrogenase 12 (all-trans/9-cis/11-cis)	0.547339793
A_23_P60990	chromosome 2 open reading frame 54	0.546613367
A_32_P209960	class II, major histocompatibility complex, transactivator	0.545976076
A_23_P145336	major histocompatibility complex, class II, DR beta 3	0.544463909
A_23_P110791	colony stimulating factor 1 receptor	0.544271695
A_23_P33723	CD163 molecule	0.54395562
A_32_P34138	family with sequence similarity 25, member A; family with	0.542835843
A_24_P246626		0.542410841
A_24_P788878	family with sequence similarity 148, member B	0.542380347
A_23_P309281	insulin-like 3 (Leydig cell)	0.542332558
A_24_P673968	tetratricopeptide repeat domain 22	0.540373606
A_23_P53417	protein phosphatase 1, regulatory (inhibitor) subunit 1A	0.539358313
A_24_P379233	gap junction protein, beta 3, 31kDa	0.538825057
A_23_P161439	chromosome 10 open reading frame 116	0.537731342
A_23_P124095	calmodulin-like 5	0.537367369
A_23_P257993	deoxyribonuclease I-like 3	0.537355327
A_23_P210869	cytochrome c oxidase subunit IV isoform 2 (lung)	0.537203555
A_23_P87653	keratin 6B	0.536906767
A_23_P104252	inter-alpha (globulin) inhibitor H5	0.535391259
A_23_P4773	leukocyte immunoglobulin-like receptor, subfamily B (with TM and ITIM	0.535323994
A_23_P70520	corneodesmosin	0.534614209
A_23_P139123	serpin peptidase inhibitor, clade G (C1 inhibitor), member 1	0.531653096
A_32_P220798	CD34 molecule	0.531239369
A_23_P200138	SLAM family member 8	0.529631416
A_23_P350396	corneodesmosin	0.52881659
A_23_P72487	Xg blood group	0.528327005
A_23_P66637	sarcoglycan, alpha (50kDa dystrophin-associated glycoprotein)	0.527538759
A_23_P55270	chemokine (C-C motif) ligand 18 (pulmonary and activation-regulated)	0.527036265

A_23_P76218	dermcidin	0.526888113
A_32_P103060		0.526787009
A_23_P206920	myosin, heavy chain 11, smooth muscle	0.526159499
A_24_P666035	keratin 77	0.524966356
A_32_P63162		0.523653872
A_23_P304716	hairy and enhancer of split 2 (Drosophila)	0.523103819
A_32_P175282		0.522959843
A_24_P400690		0.522873782
A_24_P94916	leukocyte specific transcript 1	0.522864572
A_24_P306994		0.522294278
A_23_P141802	serpin peptidase inhibitor, clade B (ovalbumin), member 7	0.520190923
A_23_P5654	interleukin 1 family, member 7 (zeta)	0.519138144
A_24_P49183	exonuclease 3'-5' domain containing 3	0.518918252
A_23_P27994	TYRO protein tyrosine kinase binding protein	0.516325587
A_24_P222655	complement component 1, q subcomponent, A chain	0.51502066
A_32_P393316	Rap guanine nucleotide exchange factor (GEF) 3	0.513584894
A_23_P2745	gap junction protein, beta 6, 30kDa	0.51284858
A_23_P160920	PDZK1 interacting protein 1	0.512690648
A_24_P586712	tumor protein p63 regulated 1	0.512259698
A_23_P124108	integrin, alpha M (complement component 3 receptor 3 subunit)	0.511421468
A_23_P25086	short chain dehydrogenase/reductase family 9C, member 7	0.511005857
A_23_P353524	involucrin	0.510760304
A_24_P167668	latent transforming growth factor beta binding protein 2	0.510021085
A_32_P100830		0.50843927
A_32_P3113		0.508037708
A_23_P106080	ribonuclease, RNase A family, 7	0.507928013
A_32_P356316	major histocompatibility complex, class II, DO alpha	0.506527766
A_23_P76249	keratin 6B	0.505946362
A_23_P100022	synaptic vesicle glycoprotein 2B; hypothetical protein LOC100128403	0.505672352
A_24_P255874		0.505600698
A_24_P288836	major histocompatibility complex, class II, DP beta 2 (pseudogene)	0.505129328
A_23_P114883	fibromodulin	0.504539938
A_24_P403959	ribonuclease, RNase A family, 1 (pancreatic)	0.503615698
A_24_P313576	vesicle-associated membrane protein 2 (synaptobrevin 2)	0.503213471
A_23_P254507	HOP homeobox	0.503040437

A_23_P324538	late cornified envelope 3B	0.502913094
A_23_P22444	complement factor properdin	0.501950155
A_23_P31006	major histocompatibility complex, class II, DR beta 5	0.501903057
A_23_P39856	interleukin 1 family, member 5 (delta)	0.501480761
A_23_P142533	collagen, type III, alpha 1	0.501340861
A_24_P839530		0.500676799
A_32_P149735		0.500649348
A_23_P308954	basic helix-loop-helix domain containing, class B, 9	0.499461043
A_23_P382607	wingless-type MMTV integration site family, member 4	0.498803294
A_24_P716394		0.498698253
A_23_P72157	major facilitator superfamily domain containing 7	0.498275568
A_24_P347310	26 serine protease	0.498011841
A_32_P140139	coagulation factor XIII, A1 polypeptide	0.497729793
A_24_P484797	cell death-inducing DFFA-like effector c pseudogene	0.497234737
A_23_P154627	teashirt zinc finger homeobox 2	0.497202604
A_23_P53018	HRAS-like suppressor family, member 5	0.496386593
A_23_P63521	late cornified envelope 2C	0.495792755
A_23_P105212	thyroid hormone responsive (SPOT14 homolog, rat)	0.495480786
A_32_P47166		0.494551469
A_24_P348203	leucine rich repeat containing 8 family, member E	0.494041637
A_23_P152406	calpain, small subunit 2	0.493101271
A_32_P169550		0.491097857
A_32_P1516		0.491050617
A_32_P68055		0.489401672
A_32_P108592		0.488826109
A_23_P10506	prostaglandin D2 synthase, hematopoietic; prostaglandin D2 synthase 21kDa (brain)	0.488693236
A_24_P75190	hemoglobin, delta	0.488063311
A_23_P131089	KN motif and ankyrin repeat domains 3	0.48797939
A_23_P149946	protocadherin 21	0.487557507
A_24_P354800	major histocompatibility complex, class II, DO alpha	0.487401985
A_32_P99492		0.48669043
A_32_P218707		0.48664017
A_32_P144999		0.486245134
A_23_P150064	multimerin 2	0.486182266
A_32_P47538		0.486035921
A_24_P289795	chromosome 1 open reading frame 46	0.485993071
A_23_P97606	glutathione S-transferase mu 5	0.485831935
A_23_P46131	glycine/arginine rich protein 1	0.485465889

A_23_P139028	POU class 2 homeobox 3	0.484047091
A_24_P165423	retinol binding protein 7, cellular	0.483988659
A_23_P106362	aquaporin 9	0.483544056
A_24_P33217		0.483538292
A_23_P146274	stathmin-like 2	0.483102182
A_23_P42588	GTPase, IMAP family member 5	0.482692317
A_24_P207828	late cornified envelope 2B	0.482465261
A_23_P19510	major histocompatibility complex, class II, DQ beta 2	0.48234131
A_23_P127824	troponin T type 3 (skeletal, fast)	0.482280834
A_23_P331813	zinc finger protein 687	0.481562308
A_32_P166921		0.481149738
A_24_P561165		0.481142411
A_23_P131899	syndecan binding protein (syntenin) 2	0.48094421
A_32_P524614	chromosome 17 open reading frame 74	0.480851715
A_23_P501010	collagen, type XVII, alpha 1	0.480505681
A_24_P147461	serpin peptidase inhibitor, clade B (ovalbumin), member 8	0.480349967
A_24_P80204	mal, T-cell differentiation protein-like	0.480209227
A_23_P38876	lipase, hormone-sensitive	0.479828918
A_24_P343233	major histocompatibility complex, class II, DR beta 4; major	0.478994242
A_32_P87697	major histocompatibility complex, class II, DR alpha	0.478697491
A_24_P291658	alcohol dehydrogenase 1B (class I), beta polypeptide; alcohol dehydrogenase	0.477764862
A_23_P74920	late cornified envelope 2B	0.477712286
A_23_P11644	small proline-rich protein 2D	0.477598074
A_23_P501713	interleukin 1 family, member 10 (theta)	0.477529607
A_23_P38696	desmocollin 1	0.477134617
A_24_P243528	major histocompatibility complex, class II, DP alpha 1	0.476436076
A_24_P153035	keratin 17 pseudogene 1	0.476376661
A_23_P161727	heat shock 27kDa protein 2	0.476241491
A_24_P379165	forkhead box O4	0.476090574
A_23_P154358	prominin 2	0.475571662
A_23_P98304	anoctamin 1, calcium activated chloride channel	0.475463358
A_23_P65618	transglutaminase 1 (K polypeptide epidermal type I, protein-glutamine-gamma-glutamyltransferase)	0.475461118
A_23_P90710	desmin	0.475221685
A_23_P500206	interleukin 17 receptor E	0.473403615
A_23_P34700	troponin T type 2 (cardiac)	0.472963735
A_24_P17870	HLA complex P5	0.471662271
A_23_P159893	chordin-like 1	0.471511673

A_23_P91334	heat shock 70kD protein 12B	0.471438512
A_23_P55749	collagen, type V, alpha 3	0.469756417
A_32_P122590	hypothetical protein LOC284242	0.468875911
A_32_P9348		0.468467375
A_23_P168388	GTPase, IMAP family member 8	0.468121544
A_23_P6321	claudin 5	0.467738848
A_23_P146946	cystatin E/M	0.467538576
A_23_P68436	WAP four-disulfide core domain 12	0.467324002
A_32_P143980		0.467168299
A_24_P935491	collagen, type III, alpha 1	0.467023519
A_24_P10233	death-associated protein kinase 2	0.466904832
A_23_P217114	aminolevulinate, delta-, dehydratase	0.466659688
A_23_P308058	tumor suppressor candidate 5	0.465680715
A_23_P139198	lectin, galactoside-binding, soluble, 12	0.465042061
A_23_P106906	periplakin	0.464922604
A_24_P64442		0.464905927
A_24_P56833	hydroxysteroid (17-beta) dehydrogenase 1	0.464175378
A_24_P225534	rhomboid, veinlet-like 2 (Drosophila)	0.464047537
A_24_P578437		0.463514161
A_23_P258504	T cell receptor alpha constant; T cell receptor alpha	0.462885118
A_23_P107963	fucosyltransferase 1 (galactoside 2-alpha-L-fucosyltransferase, H blood group)	0.462544757
A_24_P326511	sorbin and SH3 domain containing 1	0.462506136
A_24_P343621	enoyl Coenzyme A hydratase domain containing 3	0.462313359
A_32_P161755		0.461296566
A_23_P201295	castor zinc finger 1	0.461130022
A_23_P74609	G0/G1switch 2	0.460548305
A_23_P29551	transmembrane protein 40	0.460211493
A_23_P35205	RCAN family member 3	0.458875449
A_23_P165028	death-associated protein kinase 3	0.45882097
A_32_P48256		0.458644448
A_23_P417383	aspartic peptidase, retroviral-like 1	0.458627282
A_32_P172198		0.458325635
A_23_P19176	chromosome 5 open reading frame 46	0.457656987
A_24_P323148	LY6/PLAUR domain containing 5	0.456897676
A_23_P42168	MyoD family inhibitor	0.456548507
A_24_P925505	CD36 molecule (thrombospondin receptor)	0.455881858
A_32_P211276		0.455621344
A_23_P71270	alpha-2-glycoprotein 1, zinc-binding pseudogene 1; alpha-2-glycoprotein 1, zinc-binding	0.45551964
A_23_P103932	Gardner-Rasheed feline sarcoma viral (v-fgr) oncogene homolog	0.455512345
A_23_P90436	cartilage oligomeric matrix protein	0.455323282

A_32_P48825	keratin 72	0.45500065
A_24_P283189	CD14 molecule	0.454875246
A_24_P389916	leucine rich repeat containing 32	0.454854068
A_24_P369232	coiled-coil domain containing 3	0.45481449
A_23_P108062	lectin, galactoside-binding, soluble, 7; lectin, galactoside-binding, soluble, 7B	0.454632261
A_32_P213306		0.454361997
A_32_P109532		0.454170341
A_23_P411321	late cornified envelope 1F	0.45277169
A_23_P168909	zinc finger protein, multitype 2	0.452642707
A_23_P111583	CD36 molecule (thrombospondin receptor)	0.452507617
A_23_P252082	transmembrane protein 176A	0.451855855
A_32_P20492		0.451476236
A_24_P916164		0.451249071
A_23_P128783	E2F-associated phosphoprotein	0.451210909
A_23_P12292	chromosome 1 open reading frame 116	0.451166039
A_23_P163166	leukotriene B4 receptor 2	0.450208555
A_23_P104881	roundabout homolog 4, magic roundabout (Drosophila)	0.449801047
A_32_P207390		0.449621491
A_23_P77389		0.44936256
A_23_P368729	transcription factor EB	0.449179305
A_23_P353478	class II, major histocompatibility complex, transactivator	0.448936028
A_23_P92025	cell death-inducing DFFA-like effector c	0.448552248
A_24_P156748	solute carrier family 30 (zinc transporter), member 2	0.448134286
A_24_P128977	GTPase activating protein (SH3 domain) binding protein 2	0.447824631
A_32_P168973	similar to Keratin, type I cytoskeletal 16 (Cytokeratin-16) (CK-16)	0.44756997
A_23_P67799	transmembrane protein 37	0.446438525
A_32_P224498		0.446266367
A_24_P382579	oxytocin, prepropeptide	0.44613732
A_32_P219126		0.44541224
A_23_P58557	laeverin	0.445335455
A_23_P321972	FYVE, RhoGEF and PH domain containing 5	0.445054443
A_23_P21092	calbindin 2	0.44466448
A_23_P23669	palmdelphin	0.443137072
A_24_P540555		0.442848193
A_23_P203267	tripartite motif-containing 29	0.4423914
A_32_P216520	WNT inhibitory factor 1	0.442205397
A_24_P265346	keratin 14	0.442170512
A_24_P213944	hepatocyte cell adhesion molecule; HEPACAM	0.44160629

	opposite strand 1	
A_23_P123732	chromosome 9 open reading frame 103	0.441334378
A_32_P45297		0.440494634
A_32_P351968	major histocompatibility complex, class II, DM beta	0.440221395
A_23_P356494	serine peptidase inhibitor, Kazal type 5	0.438706913
A_23_P402331	WAP four-disulfide core domain 5	0.438592139
A_23_P88099	MCF.2 cell line derived transforming sequence-like	0.438391586
A_23_P89691	sidekick homolog 2 (chicken)	0.437447269
A_32_P24531		0.437223132
A_23_P340338	late cornified envelope 3E	0.436898855
A_23_P19142	potassium large conductance calcium-activated channel, subfamily M, beta member	0.436567922
A_24_P384469		0.436305576
A_23_P327380	tumor protein p63	0.43585006
A_23_P255672	actin binding LIM protein family, member 2	0.434791424
A_32_P153916		0.434391434
A_24_P399490	kallikrein-related peptidase 10	0.434360328
A_23_P209700	neuromedin U receptor 1	0.432491477
A_23_P119311	kallikrein-related peptidase 9	0.432314455
A_23_P125977	complement component 1, q subcomponent, C chain	0.432195892
A_23_P52227	growth differentiation factor 10	0.431975437
A_23_P23171	eukaryotic translation initiation factor 2C, 4	0.429896658
A_24_P104512	envoplakin	0.429794579
A_23_P211401	kringle containing transmembrane protein 1	0.429574529
A_23_P45011	protein phosphatase 1, regulatory (inhibitor) subunit 14C	0.429525298
A_24_P413470	tumor protein p73	0.429391922
A_23_P141505	C-type lectin domain family 10, member A	0.428982401
A_32_P215691		0.42807984
A_23_P126706	angiopoietin-like 1	0.427897927
A_32_P50417		0.427736905
A_24_P786912		0.427728834
A_24_P185117	Rab interacting lysosomal protein	0.42772126
A_23_P329261	potassium inwardly-rectifying channel, subfamily J, member 2	0.427646033
A_32_P35906		0.427637482
A_23_P17826	solute carrier family 5 (sodium/glucose cotransporter), member 1	0.42744726
A_32_P204903		0.427128624
A_23_P90453	keratinocyte differentiation-associated protein	0.426697491
A_23_P415510	ladinin 1	0.426690955
A_23_P147665	olfactomedin-like 1	0.426429541
A_32_P28634		0.42633541

A_32_P128391		0.426268291
A_24_P135461		0.426055935
A_32_P332320		0.425803346
A_24_P179816	solute carrier family 27 (fatty acid transporter), member 3	0.425537366
A_32_P119627		0.425288904
A_32_P179295	ribosomal protein S9; ribosomal protein S9 pseudogene 4	0.42457707
A_32_P226869		0.424229522
A_23_P48088	CD27 molecule	0.42385608
A_24_P357936		0.423672863
A_23_P405761	related RAS viral (r-ras) oncogene homolog 2; similar to	0.423406899
A_24_P7594	apolipoprotein L, 6	0.423101253
A_32_P69930	coiled-coil domain containing 96	0.423038016
A_24_P376422	similar to calcium-promoted Ras inactivator	0.423037512
A_24_P337671	G protein-coupled receptor 115	0.422542351
A_23_P217704	glycogenin 2	0.422024528
A_23_P35330	human immunodeficiency virus type I enhancer binding protein 3	0.421921351
A_23_P66767	gamma-glutamyltransferase 6	0.421664214
A_32_P210744		0.421595575
A_32_P223456	ribosomal protein S9; ribosomal protein S9 pseudogene 4	0.421592958
A_23_P46315	DENN/MADD domain containing 2C	0.42126437
A_23_P391396	early B-cell factor 3	0.420913116
A_24_P290087		0.420754229
A_23_P131990	visual system homeobox 1	0.420530665
A_32_P7602		0.420464421
A_23_P32500	stabilin 1	0.419852776
A_23_P418006	late cornified envelope 2A	0.419703963
A_23_P151895	cartilage intermediate layer protein, nucleotide pyrophosphohydrolase	0.419684343
A_24_P367242	keratin associated protein 19-1	0.417581865
A_23_P345820	WD repeat and FYVE domain containing 3	0.41723426
A_24_P230570	forkhead box S1	0.417037393
A_24_P121891	fuzzy homolog (Drosophila)	0.416774921
A_23_P207967	KIAA0427	0.416700609
A_32_P93036		0.416292699
A_32_P58407	potassium voltage-gated channel, Shal-related subfamily, member 3	0.414984509
A_24_P363896	collagen, type XXVII, alpha 1	0.414676102
A_23_P201940	leiomodin 1 (smooth muscle)	0.414058743
A_32_P38286	t-complex-associated-testis-expressed 3	0.413729248

A_24_P887092		0.413673803
A_23_P315241	actinin, alpha 4	0.41355043
A_23_P328600	solute carrier family 24 (sodium/potassium/calcium exchanger), member 6	0.412910653
A_23_P56978	PTK6 protein tyrosine kinase 6	0.412847798
A_24_P310256	leucine-rich repeat LGI family, member 4	0.412622433
A_23_P156788	syntaxin 11	0.412609501
A_23_P397376	v-maf musculoaponeurotic fibrosarcoma oncogene homolog (avian)	0.412514743
A_23_P119593	epoxide hydrolase 3	0.412469956
A_24_P184295	EH-domain containing 1	0.412444483
A_23_P344421	roundabout homolog 4, magic roundabout (Drosophila)	0.412317061
A_23_P57570	alpha 1,4-galactosyltransferase	0.412226253
A_23_P305292	hypothetical LOC728264	0.41215884
A_23_P257763	Boc homolog (mouse)	0.411756414
A_23_P201854	gap junction protein, beta 3, 31kDa	0.411688988
A_24_P499481		0.411639786
A_23_P145631	GTPase, IMAP family member 6	0.411637659
A_24_P99984		0.411615764
A_32_P121234		0.411577768
A_32_P1360		0.411551196
A_32_P123729		0.410623087
A_23_P146554	prostaglandin D2 synthase, hematopoietic; prostaglandin D2 synthase 21kDa (brain)	0.410475779
A_23_P63254	stratifin	0.409575596
A_24_P113341	chromosome 19 open reading frame 12	0.40920923
A_24_P882732	keratin 17 pseudogene 1	0.409090102
A_23_P30294	cysteine dioxygenase, type I	0.408886736
A_23_P19894	aquaporin 1 (Colton blood group)	0.408448594
A_24_P599225		0.408211886
A_24_P753454	hypothetical protein LOC100132273	0.408054654
A_32_P60632		0.407691855
A_24_P79300	claudin 5	0.407402385
A_23_P4561	serpin peptidase inhibitor, clade B (ovalbumin), member 8	0.406941101
A_23_P117104	aquaporin 5	0.406923573
A_23_P71268	alpha-2-glycoprotein 1, zinc-binding pseudogene 1; alpha-2-glycoprotein 1, zinc-binding	0.406733606
A_23_P376488	tumor necrosis factor (TNF superfamily, member 2)	0.406464176
A_23_P131909	chromosome 20 open reading frame 141	0.406403387
A_23_P43337	FRAS1 related extracellular matrix 1	0.406009086

A_23_P411993	inter-alpha (globulin) inhibitor H5	0.405859433
A_24_P41794	psoriasis susceptibility 1 candidate 2	0.405855498
A_23_P21548	Rho GTPase activating protein 10	0.405644541
A_23_P47728	microtubule-associated protein 6	0.405495987
A_24_P315885		0.405408667
A_24_P582241		0.405328083
A_24_P45446	guanylate binding protein 4	0.405202301
A_32_P107617	surfactant protein D	0.404934882
A_24_P127719	v-maf musculoaponeurotic fibrosarcoma oncogene homolog A (avian)	0.404783653
A_23_P58137		0.40450314
A_23_P395054	annexin A8-like 2	0.404390558
A_23_P77908	solute carrier family 47, member 2	0.404332929
A_23_P368101	SAP30-like	0.404295154
A_23_P34644	Fc fragment of IgG, low affinity IIb, receptor (CD32);	0.404290196
A_24_P196592	matrix metallopeptidase 28	0.404212502
A_23_P117912	ras homolog gene family, member V	0.404118065
A_23_P61057	interleukin 16 (lymphocyte chemoattractant factor)	0.404091022
A_24_P107303	interleukin 10 receptor, alpha	0.403861395
A_23_P400378	G protein-coupled bile acid receptor 1	0.403853385
A_24_P842872		0.402691044
A_24_P48187	CASK interacting protein 2	0.402264577
A_32_P83784	ArfGAP with RhoGAP domain, ankyrin repeat and PH domain	0.402245005
A_32_P213330	Rho-guanine nucleotide exchange factor	0.401992845
A_32_P176344		0.401912408
A_23_P360215	polymerase (DNA-directed), delta 4	0.401740889
A_23_P256131	hairless homolog (mouse)	0.401660034
A_23_P210708	signal-regulatory protein alpha	0.401624127
A_24_P264059		0.401423452
A_24_P324141	mucin 5B, oligomeric mucus/gel-forming	0.400161088

Table 3. GORILLA rank ordered GO gene set enrichment analysis

**Immunity**	**p-value**	**q-value**
immune response	1.44E-08	2.35E-05
complement activation	2.10E-06	2.28E-03
regulation of antigen processing and presentation	8.96E-06	7.80E-03
regulation of cytokine secretion	1.99E-05	1.44E-02
peptide antigen assembly with MHC class II protein complex	6.02E-05	3.28E-02
regulation of lymphocyte proliferation	6.88E-05	3.45E-02
regulation of leukocyte cell-cell adhesion	8.22E-05	3.70E-02
regulation of mononuclear cell proliferation	8.33E-05	3.63E-02
positive regulation of antibacterial peptide production	9.68E-05	3.16E-02
regulation of interferon-gamma production	1.00E-04	3.19E-02
interleukin-6 secretion	1.79E-04	3.76E-02
regulation of tumor necrosis factor production	2.21E-04	4.12E-02
regulation of T cell activation	2.15E-04	4.20E-02
**Epidermal function**		
keratinization	9.04E-16	5.91E-12
keratinocyte differentiation	4.88E-13	2.12E-09
epidermal cell differentiation	2.53E-11	8.25E-08
epidermis development	6.82E-11	1.78E-07
epithelium development	7.16E-10	1.56E-06
epithelial cell differentiation	1.28E-08	2.40E-05
**Cellular processes**		
peptide cross-linking	6.52E-17	8.52E-13
tissue development	8.58E-07	1.12E-03
protein activation cascade	1.34E-06	1.59E-03
regulation of cell activation	1.63E-05	1.26E-02
regulation of cell-cell adhesion	2.97E-05	2.04E-02
regulation of homotypic cell-cell adhesion	4.21E-05	2.50E-02
regulation of protein secretion	4.96E-05	2.81E-02
protein-carbohydrate complex assembly	1.26E-04	3.65E-02
regulation of cyclic nucleotide biosynthetic process	1.96E-04	3.89E-02
protein-carbohydrate complex subunit organization	1.26E-04	3.82E-02
**Cell metabolism**		
glycerophosphate shuttle	8.93E-05	3.65E-02
NADH oxidation	8.93E-05	3.54E-02
regulation of lipoprotein oxidation	1.79E-04	4.17E-02
negative regulation of glutamine transport	1.79E-04	4.09E-02
regulation of glutamine transport	1.79E-04	4.02E-02
cellular response to L-ascorbic acid	1.79E-04	3.95E-02
positive regulation of fat cell apoptotic process	1.79E-04	3.89E-02

regulation of fat cell apoptotic process	1.79E-04	3.82E-02
regulation of glucagon secretion	1.79E-04	3.70E-02
negative regulation of glucagon secretion	1.79E-04	3.65E-02
regulation of lipoprotein lipid oxidation	1.79E-04	3.59E-02
positive regulation of fat cell differentiation	2.17E-04	4.11E-02

Collagen organization

regulation of collagen biosynthetic process	1.31E-04	3.73E-02
collagen fibril organization	1.46E-04	3.97E-02
regulation of collagen metabolic process	2.69E-04	4.75E-02

Table 4. Differentially expressed genes used to define APN pathway score

Gene symbol	Agilent ID	Gene Name
**APN up-regulated genes**		
Ccr7	A_23_P343398	chemokine (C-C motif) receptor 7
Serpine1	A_23_P359111	SERPINE1 mRNA binding protein 1
Adora2b	A_23_P55477	hypothetical LOC100131909; adenosine A2b receptor
Slamf1	A_23_P62647	signaling lymphocytic activation molecule family member 1
Il6	A_23_P71037	interleukin 6 (interferon, beta 2)
Cxcl1	A_23_P7144	chemokine (C-X-C motif) ligand 1 (melanoma growth stimulating activity, alpha)
Il12b	A_23_P7560	interleukin 12B (natural killer cell stimulatory factor 2, cytotoxic lymphocyte maturation factor 2
Il1b	A_23_P79518	interleukin 1, beta
Sykb	A_23_P9255	spleen tyrosine kinase
Unc93a	A_24_P103434	unc-93 homolog A (C. elegans)
Hspa1a	A_24_P123616	heat shock 70kDa protein 1A; heat shock 70kDa protein 1B
Gna13	A_24_P150874	guanine nucleotide binding protein (G protein), alpha 13
Cxcl3	A_24_P183150	chemokine (C-X-C motif) ligand 3
Ptgs2	A_24_P250922	prostaglandin-endoperoxide synthase 2 (prostaglandin G/H synthase and cyclooxygenase
Dusp2	A_24_P37409	dual specificity phosphatase 2
Tnfsf9	A_24_P5856	tumor necrosis factor (ligand) superfamily, member 9
**APN down-regulated genes**		
Timp2	A_23_P107401	TIMP metallopeptidase inhibitor 2
Mrc1	A_23_P12746	mannose receptor, C type 1-like 1
Dusp6	A_23_P139704	dual specificity phosphatase 6
Rcbtb2	A_23_P14105	regulator of chromosome condensation (RCC1) and BTB (POZ) domain containing protein 2
Clec10a	A_23_P141505	C-type lectin domain family 10, member A
Mfsd6	A_23_P28530	major facilitator superfamily domain containing 6
Hip1	A_23_P71033	huntingtin interacting protein 1
Gcnt1	A_23_P9232	glucosaminyl (N-acetyl) transferase 1, core 2 (beta-1,6-N-acetylglucosaminyltransferase)
Lrmp	A_23_P98910	lymphoid-restricted membrane protein
Ccr2	A_24_P11825	chemokine (C-C motif) receptor 2
Rnf150	A_24_P350589	ring finger protein 150
St6gal1	A_24_P388528	ST6 beta-galactosamide alpha-2,6-sialyltranferase 1
Tlr4	A_32_P66881	toll-like receptor 4

Table 5. Demographic and clinical characteristics of SSc patients and healthy controls providing skin biopsies for fibroblast cultures.

Study Code	Age (yrs)	Sex	Onset	Subtype	MRSS
SSc1066	21	M	Early	dcSSc	15
SSc1420	49	F	Early	dcSSc	47
SSc1517	59	M	Early	dcSSc	23
SSc1096	26	F	Late	lcSSc	5
SSc1660	65	F	Late	dcSSc	19
N900	43	F			
N901	43	F			
N1022	28	F			
HDF	n/a	n/a			
N76582	43	F			

Subjects providing skin for fibroblast culture. dcSSc, diffuse cutaneous SSc; lcSSc, limited cutaneous SSc; M, male, F, female. MRSS, modified Rodnan skin score (1-51). Controls were healthy subjects. Early onset, <2 years from first non Raynaud's symptom.
